# Predictive neuromodulation of cingulo-frontal neural dynamics in major depressive disorder using a brain-computer interface system: A simulation study

**DOI:** 10.3389/fncom.2023.1119685

**Published:** 2023-03-06

**Authors:** Hao Fang, Yuxiao Yang

**Affiliations:** ^1^Department of Electrical and Computer Engineering, University of Central Florida, Orlando, FL, United States; ^2^Ministry of Education (MOE) Frontier Science Center for Brain Science and Brain-Machine Integration, Zhejiang University, Hangzhou, Zhejiang, China; ^3^State Key Laboratory of Brain-Machine Intelligence, Zhejiang University, Hangzhou, Zhejiang, China; ^4^College of Computer Science and Technology, Zhejiang University, Hangzhou, Zhejiang, China; ^5^Department of Neurosurgery, Second Affiliated Hospital, School of Medicine, Zhejiang University, Hangzhou, Zhejiang, China

**Keywords:** closed-loop neuromodulation, deep brain stimulation, brain-computer interface, major depressive disorder, system identification, predictive control, neural dynamics

## Abstract

**Introduction:**

Deep brain stimulation (DBS) is a promising therapy for treatment-resistant major depressive disorder (MDD). MDD involves the dysfunction of a brain network that can exhibit complex nonlinear neural dynamics in multiple frequency bands. However, current open-loop and responsive DBS methods cannot track the complex multiband neural dynamics in MDD, leading to imprecise regulation of symptoms, variable treatment effects among patients, and high battery power consumption.

**Methods:**

Here, we develop a closed-loop brain-computer interface (BCI) system of predictive neuromodulation for treating MDD. We first use a biophysically plausible ventral anterior cingulate cortex (vACC)-dorsolateral prefrontal cortex (dlPFC) neural mass model of MDD to simulate nonlinear and multiband neural dynamics in response to DBS. We then use offline system identification to build a dynamic model that predicts the DBS effect on neural activity. We next use the offline identified model to design an online BCI system of predictive neuromodulation. The online BCI system consists of a dynamic brain state estimator and a model predictive controller. The brain state estimator estimates the MDD brain state from the history of neural activity and previously delivered DBS patterns. The predictive controller takes the estimated MDD brain state as the feedback signal and optimally adjusts DBS to regulate the MDD neural dynamics to therapeutic targets. We use the vACC-dlPFC neural mass model as a simulation testbed to test the BCI system and compare it with state-of-the-art open-loop and responsive DBS treatments of MDD.

**Results:**

We demonstrate that our dynamic model accurately predicts nonlinear and multiband neural activity. Consequently, the predictive neuromodulation system accurately regulates the neural dynamics in MDD, resulting in significantly smaller control errors and lower DBS battery power consumption than open-loop and responsive DBS.

**Discussion:**

Our results have implications for developing future precisely-tailored clinical closed-loop DBS treatments for MDD.

## 1. Introduction

Major depressive disorder (MDD) is one of the most disabling and costly neuropsychiatric disorders. The global prevalence of MDD is estimated to be 163 million (James et al., 2018). Moreover, more than 30% of MDD patients are treatment-resistant, meaning they do not respond to medication or psychotherapy (Rush et al., 2006; Mrazek et al., 2014). The total global annual economic burden of treatment-resistant MDD is estimated at over 900 billion US dollars based on patient-per-year cost (Mrazek et al., 2014). Deep brain stimulation (DBS) is a promising therapy for treatment-resistant MDD (Dandekar et al., 2018; Bergfeld et al., 2022; Fenoy et al., 2022; Figee et al., 2022; Sheth et al., 2022). DBS works by surgically implanting an electrode into a specific brain region and delivering electrical stimulation pulses through the electrode to regulate abnormal neural activity and thus alleviating MDD symptoms. Several DBS targets have been proposed for MDD, e.g., the subcallosal cingulate gyrus (SCG) (Mayberg et al., 2005; Lozano et al., 2008), the lateral habenula (LHb) (Sartorius et al., 2010), the ventral anterior internal capsule/ventral striatum (VC/VS) (Malone et al., 2009), the medial forebrain bundle (MFB) (Schlaepfer et al., 2013), and the orbitofrontal cortex (OFC) (Rao et al., 2018).

Current DBS treatment for MDD is mostly open-loop, meaning that a fixed pattern of stimulation is continuously delivered without guidance from real-time treatment effects. Early open-label studies have shown that open-loop DBS is promising in alleviating MDD symptoms (Mayberg et al., 2005; Lozano et al., 2008; Malone et al., 2009; Sartorius et al., 2010; Schlaepfer et al., 2013). However, more recent randomized double-blind clinical trials have shown that open-loop DBS has variable and inconsistent treatment effects among patients (Dougherty et al., 2015; Bergfeld et al., 2016; Holtzheimer et al., 2017; Ramasubbu et al., 2020). Moreover, in addition to inter-subject response heterogeneity (Figee and Mayberg, 2021), MDD symptoms and DBS effects can change dynamically over time within a patient depending on the patient's psychiatric state (Williams, 2017; Scangos et al., 2021b). Open-loop DBS delivers fixed stimulation over time and hence does not track these dynamics. Thus, open-loop DBS can suffer from imprecise regulation of symptoms, high battery power consumption, and possible side effects (Scangos et al., 2021a; Figee et al., 2022).

Personalized DBS targeting (Figee et al., 2022) and closed-loop DBS (Scangos et al., 2021a) have been proposed to improve open-loop DBS treatment for MDD. Closed-loop DBS monitors neural activity in real time, then uses a computer program to analyze the neural activity and determine the DBS pattern that can best regulate neural activity. Such a closed-loop DBS system constitutes a brain-computer interface (BCI) system for neuromodulation where the computer aims to regulate diseased brain states with therapeutic purposes (Panuccio et al., 2016; Shanechi, 2019). State-of-the-art clinical closed-loop DBS system acts in a responsive manner where a single scalar neural biomarker of mood symptoms is first identified offline. In online neuromodulation, constant stimulation is triggered whenever the real-time computed neural biomarker crosses a pre-defined threshold value. Such responsive DBS has shown promising clinical treatment results for Parkinson's disease (Rosin et al., 2011; Little et al., 2013; Priori et al., 2013; Swann et al., 2018; Gilron et al., 2021) and epilepsy (Ben-Menachem and Krauss, 2014; Morrell and Halpern, 2016) and has motivated its recent use in MDD (Scangos et al., 2021a). While responsive DBS can track MDD neural activity dynamics to some extent and has been shown to have rapid and sustained treatment effects in one pilot clinical study (Scangos et al., 2021a), it still suffers from several limitations.

First, MDD is a complex neuropsychiatric disorder that likely involves the dysfunction of a distributed network consisting of multiple limbic and frontal regions (Mayberg, 1997; Drevets, 2001; Ramirez-Mahaluf et al., 2017; Williams, 2017). Moreover, within the limbic-frontal network, altered neural spectral activity at different frequency bands—especially the θ band (3–7 Hz) and β+low γ band (30–50 Hz)—has been observed in different mood symptom states (Sani et al., 2018; Bijanzadeh et al., 2022; Xiao et al., 2023) and after DBS (Rao et al., 2018; Smart et al., 2018; Smith et al., 2022). This means that using a scalar neural biomarker computed from a single brain site and a single frequency band (Scangos et al., 2021a) may not be sufficient for achieving precise MDD symptom regulation. Second, prior studies have shown that the neural activity in response to stimulation is dynamic (Bolus et al., 2018, 2021; Crowther et al., 2019; Stiso et al., 2019; Yang et al., 2021b), meaning that the present stimulation affects not only the present neural activity but also the future temporal evolution of neural activity. The neural responses to stimulation are also state-dependent and likely to be non-linear for MDD (Scangos et al., 2021a). This suggests that the simple threshold-crossing strategy in responsive DBS may not be optimal in regulating the non-linear dynamic MDD-related neural activity. Third, depending on the pre-defined threshold value, responsive DBS may be frequently triggered, which still consumes much battery power and reduces battery life.

More advanced predictive neuromodulation methods have been proposed and tested in simulations to address dynamic neural responses in the context of Parkinson's disease (Santaniello et al., 2010; Liu et al., 2011; Su et al., 2019; Zhu et al., 2021) and epilepsy (Ehrens et al., 2015; Nagaraj et al., 2017). Such methods first use offline system identification to build a dynamic model that quantifies the stimulation effect on neural activity and then use the model to design an online feedback controller such as a proportional-integral (PI) controller to regulate neural activity (Su et al., 2019; Zhu et al., 2021). However, these methods largely regulate neural activity at a single brain site or frequency band (see Section 4). Our prior work have extended to regulate neural activity at multiple brain sites and frequency bands using a linear-quadratic-regulator (LQR) (Yang et al., 2018a, 2021b), but have only been tested in regulating linear neural dynamics. Therefore, it remains unknown if predictive neuromodulation methods can precisely regulate the non-linear and multiband spectral activity in MDD.

Here, to address the above limitations, we develop a BCI system of predictive neuromodulation for treating MDD and use a biophysically plausible non-linear model of MDD as a simulation testbed to test the system. Within the MDD-related limbic-frontal network, the reciprocal interaction between the ventral anterior cingulate cortex (vACC) and the dorsolateral prefrontal cortex (dlPFC) forms a typified subnetwork that regulates the emotion-cognition interaction in MDD (Mayberg, 1997; Fox et al., 2012). We thus adopt and adjust an established non-linear vACC-dlPFC neural mass model (Ramirez-Mahaluf et al., 2017) to simulate the multiband spectral activity in response to DBS in MDD. The BCI system consists of offline system identification and online predictive DBS. We first conduct offline system identification experiments where we use offline data to fit a dynamic input-output (IO) model that can predict the effect of DBS (input) on vACC-dlPFC multiband spectral activity (output). Next, in online neuromodulation experiments, we use the identified dynamic IO model to design a dynamic brain state estimator and a model predictive controller (MPC). The brain state estimator estimates the MDD brain state from the history of spectral activity and previously delivered DBS. The MPC uses the fitted dynamic IO model and the estimated MDD brain state to predict the DBS effects on future spectral activity and adjusts the present DBS accordingly to regulate the spectral activity to therapeutic targets with efficient DBS energy. We compare our predictive DBS method with existing open-loop and responsive DBS using the vACC-dlPFC neural mass model as the simulation testbed. We show that in offline system identification experiments, the fitted dynamic IO model can accurately predict future spectral activity from the history of DBS and spectral activity. We also show that in online neuromodulation experiments, our predictive DBS outperforms open-loop and responsive DBS in regulating non-linear and multiband MDD spectral activity, achieving significantly smaller control errors and lower DBS energy. Our results suggest that the proposed BCI system of predictive neuromodulation provides a promising computational framework for developing precisely-tailored clinical closed-loop DBS treatments for MDD.

## 2. Materials and methods

### 2.1. The cingulo-frontal neural mass model for MDD

While MDD is a complex neuropsychiatric disorder whose disease mechanism is still under investigation (Ressler and Mayberg, 2007; Drysdale et al., 2017; Lin et al., 2022), prior studies have provided evidence that MDD can involve the dysfunction of a distributed network consisting of limbic and frontal regions (Mayberg, 1997; Drevets, 2001; Williams, 2017). Among this limbic-frontal network, the reciprocal interaction between the vACC (part of the limbic system) and dlPFC (part of the frontal cortex) is hypothesized to play a critical role in regulating the emotion-cognition interaction in MDD (Mayberg, 1997; Fox et al., 2012). Therefore, we adopt an established computational vACC-dlPFC model (Ramirez-Mahaluf et al., 2017) to simulate the neural activity in MDD. We especially adjust the original model such that the output spectral power dynamics are in line with the most recent findings in spectral signatures of mood symptoms (Rao et al., 2018; Sani et al., 2018; Smart et al., 2018; Scangos et al., 2021a; Smith et al., 2022; Xiao et al., 2023). We then use the adjusted model as a simulation testbed to evaluate offline dynamic system identification methods (see Section 2.2.2) and online neuromodulation techniques (see Sections 2.2.3 and 2.2.4).

**Figure 1:**
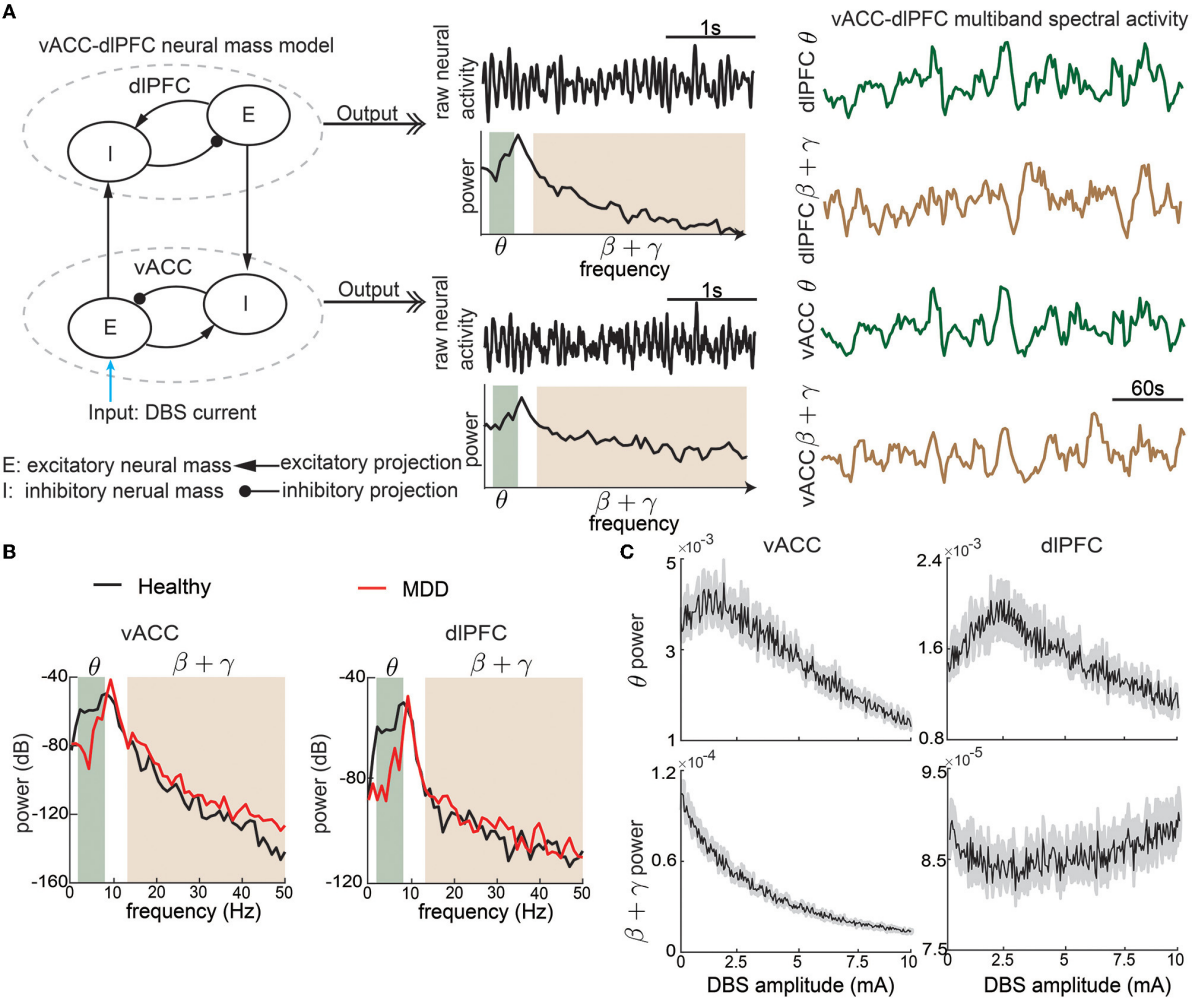
The non-linear vACC-dlPFC neural mass model for MDD. **(A)** Illustration of model construction. Left: There are two neural masses in each region, one excitatory neural mass, and one inhibitory neural mass. The four neural masses are interconnected with excitatory and inhibitory connections. DBS is modeled as the input current to the vACC excitatory neural mass. We take the model input as the DBS amplitude. Middle: The aggregate raw neural activity of each region is shown in the time domain, whose power spectrum in dB scale is shown below. θ and β + γ bands are shaded. Right: θ and β + γ power time series in each region are computed using a temporal sliding window. We take the model output as the vACC-dlPFC multiband (θ and β + γ) spectral power time series in response to DBS input. The traces show example responses to a constant DBS amplitude of 5 mA, which already exhibit dynamic patterns. **(B)** The power spectrum of vACC and dlPFC neural activity in healthy and MDD states. **(C)** The time-averaged vACC-dlPFC multiband spectral response to different DBS amplitudes. The solid line represents the mean value across 50 trials of simulation, and the gray shaded area represents the 95% confidence interval. Each averaged spectral response changes non-linearly and differently as the DBS amplitude varies.

The vACC-dlPFC model is a neural mass model (Wilson and Cowan, 1972) that consists of four neural masses—one excitatory neural mass and one inhibitory neural mass in each region. The four neural masses are interconnected with excitatory and inhibitory projections (Figure 1A) and exhibit non-linear neural dynamics that are described by the following set of ordinary differential equations


(1) $$\begin{array}{l} \{\begin{array}{l} \tau_{e}\frac{\text{d}r_{e}^{v}\left(t\right)}{\text{d}t}=-r_{e}^{v}\left(t\right)+\phi_{e}(G_{ee}f_{D}r_{e}^{v}\left(t\right)-G_{ei}r_{i}^{v}\left(t\right)+T_{e}\left(I_{e}f_{D}\right)\\ \;+g_{dbs}I_{dbs}\left(t\right)+\epsilon^{v}\left(t\right))\\ \tau_{i}\frac{\text{d}r_{i}^{v}\left(t\right)}{\text{d}t}=-r_{i}^{v}\left(t\right)+\phi_{i}\left(G_{ie}f_{D}r_{e}^{v}\left(t\right)-G_{ii}r_{i}^{v}\left(t\right)+G_{x}r_{e}^{d}\left(t\right)+I_{i}f_{D}\right)\\ \tau_{e}\frac{\text{d}r_{e}^{d}\left(t\right)}{\text{d}t}=-r_{e}^{v}\left(t\right)+\phi_{e}\left(G_{ee}r_{e}^{d}\left(t\right)-G_{ei}r_{i}^{d}\left(t\right)+I_{e}\right)\\ \tau_{i}\frac{\text{d}r_{i}^{d}\left(t\right)}{\text{d}t}=-r_{i}^{d}\left(t\right)+\phi_{i}(G_{ie}r_{e}^{d}\left(t\right)-G_{ii}r_{i}^{d}\left(t\right)+G_{x}r_{e}^{v}\left(t\right)\\ \;+I_{i}+\epsilon^{d}\left(t\right)) \end{array} \end{array}$$

Here, *t* is the continuous time variable, $r_{e}^{v}\left(t\right)$, $r_{i}^{v}\left(t\right)$, $r_{e}^{d}\left(t\right)$, $r_{i}^{d}\left(t\right)$ represent the neural activity of the vACC excitatory neural mass, vACC inhibitory neural mass, dlPFC excitatory neural mass, dlPFC inhibitory neural mass, respectively. τ_*e*_, τ_*i*_ are the time constants of excitatory and inhibitory masses, respectively. ϕ_*e*_ and ϕ_*i*_ are non-linear neural activation functions. The $r_{e}^{v}\left(t\right)$, $r_{i}^{v}\left(t\right)$, $r_{e}^{d}\left(t\right)$, $r_{i}^{d}\left(t\right)$ can be considered as the states of the ordinary differential system. The input to the vACC-dlPFC model is *I*_*dbs*_(*t*), which represents the amplitude of a DBS current pulse train in practice. *I*_*dbs*_(*t*) is modeled to directly drive the vACC excitatory neural mass. The input weight *g*_*dbs*_ is included such that *I*_*dbs*_(*t*) takes values in the practical range of [0 10]mA.

The model includes several parameters. ϕ_*i*_(·) and ϕ_*e*_(·) are non-linear functions that model the non-linear relationships between input currents and neural activity. Following the original model (Ramirez-Mahaluf et al., 2017), ϕ_*e*_(*x*) is taken as the following S-shape function


$$\begin{array}{l} \phi_{e}\left(x\right)=\{\begin{array}{ll} 0 & ,\;x<0\\ 20x^{2} & ,\;0\leq x\leq 1\\ 40\sqrt{x-0.75} & ,\;x>1 \end{array} \end{array}$$

and ϕ_*i*_(*x*) = 4ϕ_*e*_(*x*). *G*'s describe the effective connection strength between different types of neural masses. *I*_*i*_ and *I*_*e*_ are the current inputs from neurons external to the network. The selection of the above parameters is the same as the original model (Ramirez-Mahaluf et al., 2017, also see Table 1 for details). *f*_*D*_ > 1 represent the MDD state. Same as the original model, We set *f*_*D*_ = 1 for a healthy state and *f*_*D*_ = 1.25 for a severe MDD state that requires DBS treatment.

**Table 1:** Model parameters in the vACC-dlPFC neural mass model.

**Parameters**	**Values or functions**
*G*_*ee*_	0.09 s
*G*_*ie*_	0.04 s
*G*_*ei*_	0.0275 s
*G*_*ii*_	0.0075 s
*G*_*x*_	0.025 s
*I*_*e*_	0.163
*I*_*i*_	0.1
τ_*e*_	20 ms
τ_*i*_	20 ms
*g*_*dbs*_	0.02
*T*_*e*_(*x*)	4*x*−0.6
ϵ^*v*^(*t*)	N(0.005,0.002)
ϵ^*d*^(*t*)	N(0.005,0.002)

N(μ,σ) represents a normal distribution of mean μ and standard deviation σ.

The original vACC-dlPFC neural mass model focuses on investigating the temporal dynamics of raw neural activity $r_{e}^{v}\left(t\right)$, $r_{i}^{v}\left(t\right)$, $r_{e}^{d}\left(t\right)$, $r_{i}^{d}\left(t\right)$ and demonstrates that the model shows interesting non-linear dynamics that are consistent with clinical findings (Ramirez-Mahaluf et al., 2017). However, recent clinical findings on neural signatures of mood symptoms have shown that spectral power dynamics—especially in the θ (3–7 Hz) and β+low γ (13–50 Hz) bands—are key features that are related to mood symptoms (Rao et al., 2018; Sani et al., 2018; Smart et al., 2018; Scangos et al., 2021a; Bijanzadeh et al., 2022; Smith et al., 2022; Xiao et al., 2023). Therefore, we make two adjustments to the original vACC-dlPFC model such that we can simulate spectral power dynamics that are in line with the above clinical findings. Specifically, we include a linear function *T*_*e*_(·) and two stochastic noise terms ϵ^*v*^(*t*) and ϵ^*d*^(*t*) so that we can fully excite the spectral power dynamics of the model (see Table 1). We then take the output of the model as the θ and β+low γ band powers of the aggregate neural activity of the vACC neural masses $r^{v}\left(t\right)=r_{e}^{v}\left(t\right)+r_{i}^{v}\left(t\right)$ and the aggregate neural activity of the dlPFC neural masses $r^{d}\left(t\right)=r_{e}^{d}\left(t\right)+r_{i}^{d}\left(t\right)$ (Figure 1A). *r*^*v*^(*t*) and *r*^*d*^(*t*) can be regarded as the continuous electrical activity recorded by two electrodes in clinical practice. The final output of the adjusted vACC-dlPFC neural mass model is


(2) $$\begin{array}{l} \{\begin{array}{l} y_{\theta }^{v}\left(k\right)\;={\text{spectrogram}}_{\theta }\left(r^{v}\left(t\right)\right),\\ y_{\beta +\gamma }^{v}\left(k\right)={\text{spectrogram}}_{\beta +\gamma }\left(r^{v}\left(t\right)\right),\\ y_{\theta }^{d}\left(k\right)\;={\text{spectrogram}}_{\theta }\left(r^{d}\left(t\right)\right),\\ y_{\beta +\gamma }^{d}\left(k\right)={\text{spectrogram}}_{\beta +\gamma }\left(r^{d}\left(t\right)\right), \end{array} \end{array}$$

where ${\text{spectrogram}}_{\theta }\left(r^{v}\left(t\right)\right)$ represent computing the spectrogram of *r*^*v*^(*t*) by using the standard Welch method (Welch, 1967) with a sliding time window of 10 s and a step size of 2 s and then taking the total power in the θ band. The new discrete time step *k* represents the 2 s time step in computing the spectrogram. The other spectrogram operations in Equation (2) have similar meanings. We collect the above spectral power time sequence into a single output vector


(3) $$\begin{array}{l} y\left(k\right)={\left[y_{\theta }^{v}\left(k\right),y_{\beta +\gamma }^{v}\left(k\right),y_{\theta }^{d}\left(k\right),y_{\beta +\gamma }^{d}\left(k\right)\right]}^{\prime }, \end{array}$$

where ·′ represents vector and matrix transpose.

To summarize, the complete vACC-dlPFC neural mass model is described by Equations (1)–(3). We take the model input as the DBS current *I*_*dbs*_, and take the model output as the vACC-dlPFC multiband spectral power time series *y*(*k*). Different from the original model, we focus more on spectral power dynamics instead of the raw neural activity dynamics (see Section 4).

With the above setup, the vACC-dlPFC model indeed shows spectral power characteristics that are in line with clinical findings. Specifically, without DBS treatment (*I*_*dbs*_ = 0), the simulated MDD state (*f*_*D*_ = 1.25) has lower vACC θ power and higher vACC β + γ power than the simulated healthy state (*f*_*D*_ = 1) (Figure 1B, left panel). This is in line with recent findings in human intracranial electroencephalography (iEEG) studies that in some patients, cingulate θ power can decrease (Sani et al., 2018; Bijanzadeh et al., 2022) and β/γ power can increase (Sani et al., 2018; Bijanzadeh et al., 2022; Xiao et al., 2023) when mood symptoms become worse. This is consistent with the DBS treatment effects where cingulate θ power can increase (Smith et al., 2022) and β/γ power can decrease after DBS (i.e., improved mood symptoms) (Smart et al., 2018). Similar trends hold in the simulated dlPFC power (Figure 1B, right panel), which is also consistent with recent findings in human iEEG that in some patients, frontal θ power can decrease and β/γ power can increase when mood symptoms become worse (Sani et al., 2018).

To provide a rough idea of the non-linear dynamic input-output relationship between the DBS *I*_*dbs*_ and the spectral activity *y*(*k*), we first fix *I*_*dbs*_ at 5 mA and qualitatively investigate the output spectral activity *y*(*k*). Figure 1A shows that the output spectral activity *y*(*k*) already changes dynamically over time with the simple constant input. We then sweep *I*_*dbs*_ from 0 to 10 mA and show how the spectral power averaged over time changes as functions of *I*_*dbs*_ (Figure 1C). We see that there exist complex and different non-linear relationships for each element in *y*(*k*). The above facts suggest that precise regulation of the vACC-dlPFC multiband spectral activity using DBS is a challenging task. Subsequently, we will show how the current open-loop and responsive DBS methods fail to precisely regulate vACC-dlPFC multiband spectral activity and how we design predictive BCI DBS methods that can address the challenge.

### 2.2. BCI system design for predictive neuromodulation

#### 2.2.1. Overview of the BCI system of predictive neuromodulation

**Figure 2:**
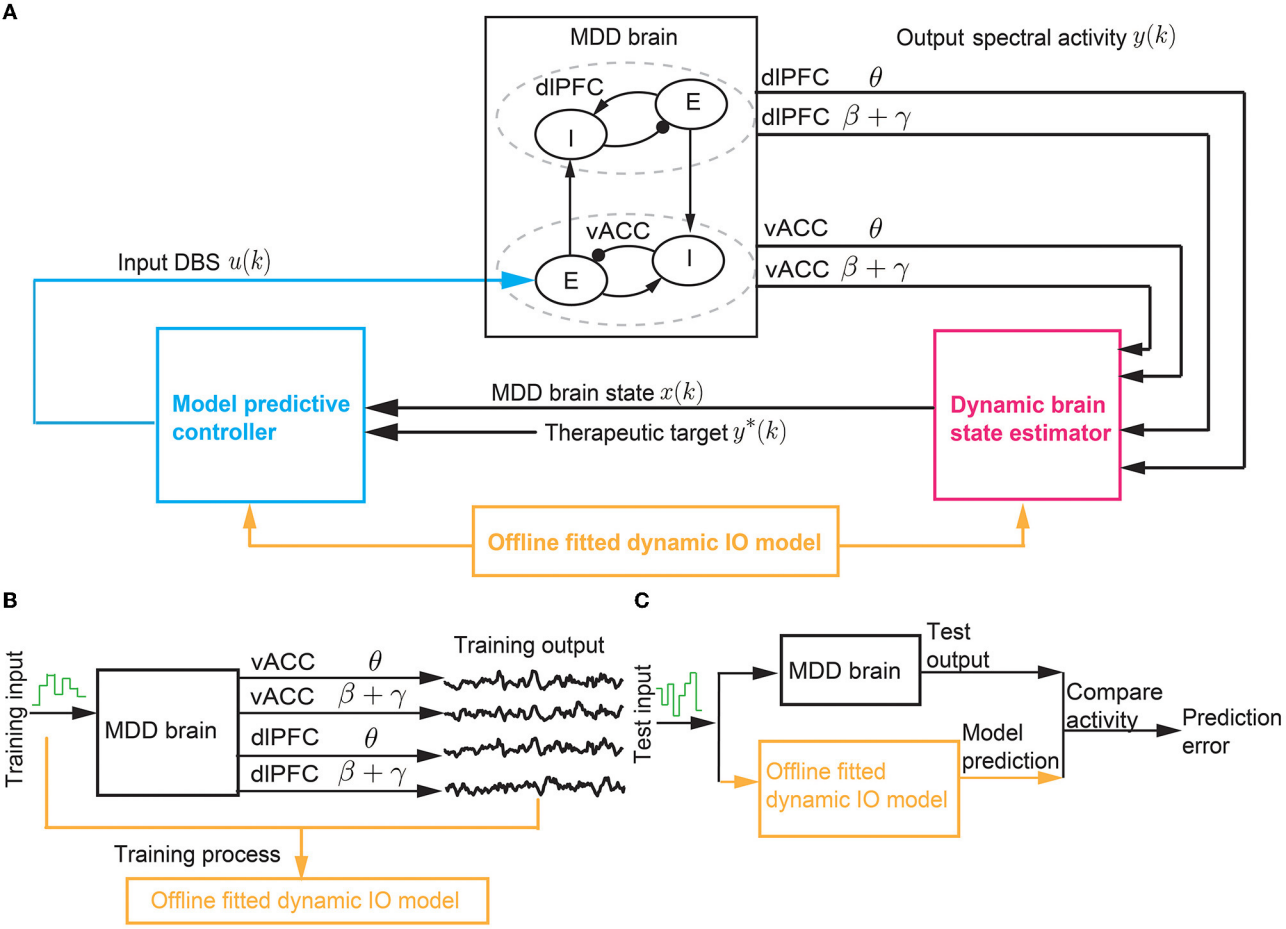
The BCI system of predictive neuromodulation. **(A)** The online BCI system consists of the dynamic brain state estimator and the model predictive controller. The vACC-dlPFC neural mass model simulates the MDD brain. The estimator and controller constitute the “computer”. The design of the estimator and controller depends on a dynamic IO model fitted in offline system identification [see **(B, C)**]. **(B)** The training process in offline dynamic system identification. **(C)** The test process in offline dynamic system identification. * stands for therapeutic target.

The BCI system aims to use predictive DBS to regulate the vACC and dlPFC θ and β + γ powers in the MDD state to follow therapeutic target values with minimum DBS energy (Figure 2A). The therapeutic target values are taken as the vACC and dlPFC θ and β + γ powers in the healthy state. Predictive DBS is a closed-loop neuromodulation system. The real-time observed spectral activity, i.e., the vACC and dlPFC θ and β + γ power time series, is fed into a dynamic brain state estimator, which aggregates the past DBS input and the past vACC and dlPFC θ and β + γ powers to estimate the present MDD brain state. The dynamic nature of the brain state estimator helps address the multiband spectral power dynamics in the vACC-dlPFC neural mass model. Then, the estimated MDD brain state is used as feedback by a model predictive controller (MPC) to adjust the DBS amplitude to optimally regulate the output neural activity to follow therapeutic targets with minimum DBS energy. The feedback mechanism in MPC helps address the non-linearity in the vACC-dlPFC neural mass model.

The closed-loop neuromodulation system constitutes a BCI where the vACC-dlPFC neural mass model acts as the “MDD brain” and the dynamic brain state estimator and MPC act as the “computer”. For simplicity, we use the term “MDD brain” to denote the complete vACC-dlPFC neural mass model in Equations (1)–(3). The MDD brain and the “computer” interacts in closed loop as described above. The “computer” does not have explicit knowledge of the ground-truth vACC-dlPFC model of the MDD brain. Rather, the “computer” can only obtain the output neural activity generated by the MDD brain. To design the dynamic brain state estimator and MPC, we identify a simplified linear dynamic input-output (IO) model to describe the IO dynamics of the MDD brain. The identification of the simplified linear dynamic model based only on IO datasets collected offline prior to online neuromodulation (Figures 2B, C).

We note that in our BCI framework in Figure 2, we use a continuous-time neural mass model (1) to simulate the state of the “MDD brain”. We use the standard numerical solver (ode45) in MATLAB to solve the continuous-time neural mass model with a discretization time step of 0.1ms, which gives discrete solutions of the neural mass states *r*^*d*^(*t*), *r*^*v*^(*t*). Then, we apply the standard Welch method on the discrete-time neural mass states to compute the corresponding discrete spectrograms with a discretization time step of 2 s, which gives the discrete-time spectral power time series *y*(*k*) (see Equation 2). Here, the discretization time step *k* represents multiples of 2 s. *y*(*k*) is then used as the feedback signal by the “computer”. We thus design the entire “computer”, i.e., the brain state estimator and MPC, with the discretization time step *k*. Such discretization follows the conventional signal processing steps in BCIs using real-world continuous-time brain signals (Liu et al., 2011; Yang et al., 2018a; Su et al., 2019). The overall BCI system includes offline dynamic system identification and online predictive DBS as summarized in Algorithm 1. In the next three sections, we expand on the details of dynamic system identification, the dynamic brain state estimator design, and the MPC design.

**Algorithm 1:**
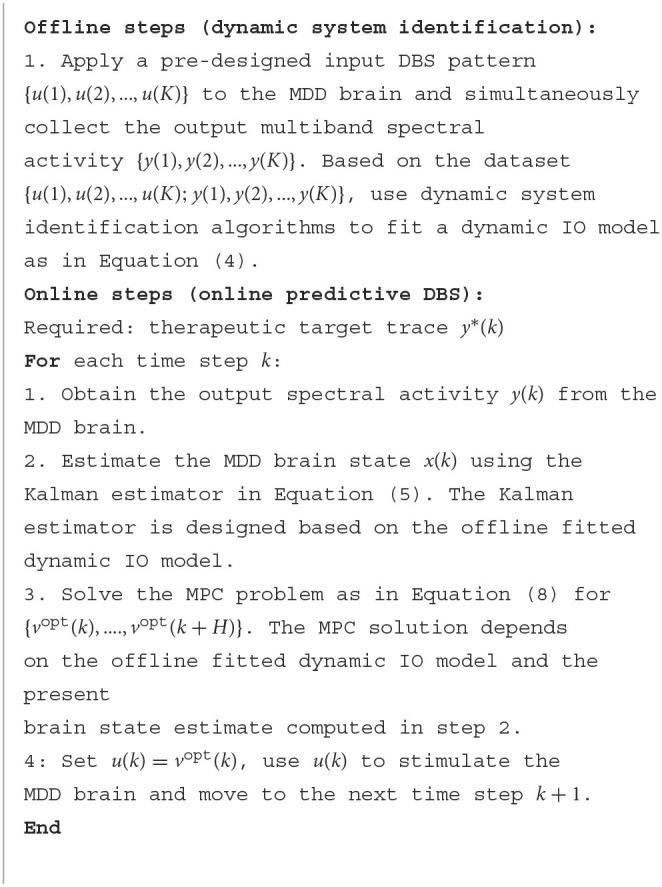
BCI for predictive neuromodulation.

#### 2.2.2. Dynamic system identification

The design of the dynamic brain state estimator and the MPC requires a dynamic model that describes the IO relationship of the MDD brain. Motivated by our prior work (Yang et al., 2018a, 2021b; Fang and Yang, 2021, 2022), we build a simplified linear state-space model


(4) $$\begin{array}{l} \{\begin{array}{l} x\left(k+1\right)=Ax\left(k\right)+Bu\left(k\right)+w\left(k\right),\\ y\left(k\right)\;=Cx_{t}+v\left(k\right). \end{array} \end{array}$$

Here, *k* is the discrete time step. $x\left(k\right)\in {\mathbb{R}}^{n_{x}}$ is a multi-dimensional hidden state that stands for the MDD brain state. *u*(*k*) ∈ ℝ represents the amplitude of the input DBS, i.e., the input *I*_*dbs*_ to the MDD brain (see Equation 1). $y\left(k\right)\in {\mathbb{R}}^{n_{y}}$ represents the vACC and dlPFC θ and β + γ power time series, i.e., the multiband output of the MDD brain (see Equations 2 and 3). *w*(*k*) and *v*(*k*) are noise terms that represent modeling errors and external disturbances. *w*(*k*) and *v*(*k*) are modeled as white Gaussian noise with zero mean and a joint covariance matrix $E\left[\left(\begin{array}{c} w_{i}\\ v_{j} \end{array}\right)\left(w_{i}^{\prime }\;v_{j}^{\prime }\right)\right]=\left(\begin{array}{cc} Q & S\\ S^{\prime } & R \end{array}\right)\delta_{ij}$with δ_*ij*_ = 1 if *i* = *j* and 0 otherwise, *E*[·] denoting the expectation operator.

The model parameters in Equation (4) are the matrices *A, B, C, Q, S, R*. The model parameters need to be fitted from IO data. We use a typical dynamic system identification method developed in our prior work (Yang et al., 2018a, 2021b). Specifically, prior to online neuromodulation, we conduct a separate offline system identification experiment where we apply a pre-designed DBS pattern {*u*(1), *u*(2), ..., *u*(*K*)} to the MDD brain and collect the output spectral activity {*y*(1), *y*(2), .., *y*(*K*)}. ({·} represents the collection of variables). The total training time step is denoted by *K*. Here, the input DBS pattern is pre-designed to be a random time-series, where each *u*(*k*) is generated by an independent uniform distribution over [0 10] mA. We then use the well-known subspace identification method N4SID (Van Overschee and De Moor, 2012) to fit *A, B, C, Q, S, R* from the training IO dataset {*u*(1), *u*(2), ..., *u*(*K*);*y*(1), *y*(2), .., *y*(*K*)} (see Figure 2B).

To test the fitted model, we apply a new random input to the MDD brain and collect the test IO dataset {*u*(1), *u*(2), ..., *u*(*J*);*y*(1), *y*(2), .., *y*(*J*)}. The total test time step is denoted by *J*. For each test time step 1 ≤ *j*_test_ ≤ *J*, we evaluate the fitted model in terms of their ability to use the past and present input and output data {*u*(1), *u*(2), ..., *u*(*j*_test_);*y*(1), *y*(2), .., *y*(*j*_test_)} to predict the future spectral activity *y*(*j*_test_ + 1) (see Section 2.3 for details).

#### 2.2.3. Dynamic brain state estimator

The dynamic brain state estimator aims to use the past and present input and output data to estimate the present MDD brain state. The MDD brain state is represented by the hidden state *x*(*k*) in the dynamic IO model (4). From the dynamic IO model fitted from offline system identification, we can derive a Kalman estimator to estimate *x*(*k*) from the past and present input and output data {*u*(1), *u*(2), ..., *u*(*k*);*y*(1), *y*(2), .., *y*(*k*)}. The Kalman estimator takes the following recursive form


(5) $$\begin{array}{l} \{\begin{array}{l} {\hat{x}}_{p}\left(k\right)=A\hat{x}\left(k-1\right)+Bu\left(k-1\right),\\ \hat{x}\left(k\right)\;={\hat{x}}_{p}\left(k\right)+L\left(y\left(k\right)-C{\hat{x}}_{p}\left(k\right)\right), \end{array} \end{array}$$

where ${\hat{x}}_{p}\left(k\right)$ is the Kalman prediction of *x*(*k*), $\hat{x}\left(k\right)$ is the Kalman estimation of *x*(*k*), and *L* is the Kalman gain. The Kalman gain *L* is a function of the model parameters *A, C, Q, S, R* and is computed from


(6) $$\begin{array}{l} L=PC^{\prime }R^{-1}-PC^{\prime }{\left(CPC^{\prime }+R\right)}^{-1}CPC^{\prime }R^{-1}, \end{array}$$

where *P* is obtained from solving the algebraic Riccati equation (Bertsekas, 2012) *P* = *APA*′ + *Q* − (*APC*′ + *S*)(*CPC*′ + *R*)^−1^(*CPA*′ + *S*). We note that by plugging the top prediction equation into the bottom update equation in (5), the Kalman estimator can also be compactly written as a single recursion equation for $\hat{x}\left(k\right)$


(7) $$\begin{array}{l} \hat{x}\left(k\right)=A\hat{x}\left(k-1\right)+Bu\left(k-1\right)+L\left(y\left(k\right)-C\left(A\hat{x}\left(k-1\right)+Bu\left(k-1\right)\right)\right). \end{array}$$

#### 2.2.4. Model predictive controller

Although MPC has been widely used in many modern control applications (Mayne, 2014), its application in neuromodulation has not drawn as much attention as simpler methods such as responsive control (e.g., Scangos et al., 2021a), PI control (e.g., Su et al., 2019), and LQR control (e.g., Yang et al., 2018a). MPC has the advantage of being predictive of future control effects, and can explicitly take account of safety constraints of input and states. Therefore, MPC is especially suited for implementing predictive neuromodulation.

Specifically, at each time step *k*, MPC solves the following finite-horizon predictive control problem


(8) $$\begin{array}{l} \underset{\text{subject to}}{\begin{array}{c} \text{minimize}\\ \left\{v\left(k\right),....,v\left(k+H\right)\right\} \end{array}}\;\overset{H}{\underset{h=1}{\sum }}||y\left(k+h\right)-y^{*}\left(k+h\right){||}^{2}+\\ \;\lambda ||v\left(k+h-1\right){||}^{2}\\ \;z\left(k+h+1\right)=Az\left(k+h\right)+Bv\left(k+h\right),\\ \;y\left(k+h\right)=Cz\left(k+h\right),\\ \;z\left(k\right)=\hat{x}\left(k\right),\\ \;0\leq v\left(k+h\right)\leq 10,\\ \;\forall 0\leq h\leq H \end{array}$$

where λ is a design parameter to balance regulation performance and energy saving. In the cost function, we aim to adjust the input variables {*v*(*k*), ...., *v*(*k* + *H*)} in the future *H* time steps for achieving two goals. First, to regulate the output *y*(*k* + *h*) to follow the predefined therapeutic target y^*^(*k* + *h*) for MDD treatment; thus we have the first penalty term. Second, to use minimal input DBS energy for saving the battery power of the DBS device; thus we have the second penalty term. In the constraints, we have a dynamic constraint among the control variables {*v*(*k*), ...., *v*(*k* + *H*)} and output variables {*y*(*k*), ...., *y*(*k* + *H*)} as specified by our offline fitted dynamic IO model in Equation (4). *z*(*k* + *h*) is an intermediate variable that describes the dynamics in predictive control. The initial condition at *h* = 0, i.e., *z*(*k*), is taken as the brain state estimate at time *k* as obtained from the Kalman estimator (Equation 7), i.e., $z\left(k\right)=\hat{x}\left(k\right)$. The use of the dynamic constraint enables MPC to predict future control effects and thus optimizes the present and future control variables for achieving optimal regulation. Finally, the constraint 0 ≤ *v*(*k* + *h*) ≤ 10 ensures the DBS amplitude is always within the clinically safe range of [0 10] mA.

It is well-known that the MPC problem (Equation 8) is a convex optimization problem (Camacho and Alba, 2013). At each time step *k*, the MPC problem (Equation 8) is solved by standard convex optimization methods (Boyd et al., 2004) with λ = 0.01. The optimal solution gives the present and future control variables {*v*^opt^(*k*), ...., *v*^opt^(*k* + *T*)}. We then set the actual DBS amplitude *u*(*k*) at the present time step as the first optimal solution variable *v*^opt^(*k*). Next, we move to the next time step *k* + 1, formulate and solve a new MPC problem, and set *u*(*k* + 1) as the first solution variable *v*^opt^(*k* + 1) in the new MPC problem. We iterate the above process till the end of the online neuromodulation process.

### 2.3. Simulation experiments, performance measures, and statistical tests

We conduct comprehensive simulation experiments to test the BCI system, including the offline dynamic system identification and online predictive DBS methods. All simulations are implemented in MATLAB2020b. In all experiments, when generating neural activity, the vACC-dlPFC neural mass model in Equation (1) is solved by the standard numerical ordinary differential equation solver ode45.

#### 2.3.1. Offline dynamic system identification experiments

In offline dynamic system identification experiments, we run 50 trials of training and test. In each training trial, the total number of training time step *K* is set to be 1500. In each test trial, the total number of test time steps *J* is set to 1500. The random input DBS pattern is generated independently in each trial. We fit a dynamic IO model (4) in the training set. In the test set, for each test time step *k*, we test the fitted model in terms of its ability to use the past and present data {*u*(1), *u*(2), ..., *u*(*k*);*y*(1), *y*(2), .., *y*(*k*)} to predict the future spectral activity *y*(*k* + 1). To illustrate the dynamic property of the model, we implement four types of prediction.

1. Only using the DBS input of the present time step, i.e., *u*(*k*), to predict *y*(*k* + 1). This prediction is a static prediction without considering the spectral activity dynamics and can be derived from a special case of the fitted IO model (4) with *A* = 0. The predictor can be written as
  (9) $$\begin{array}{l} \hat{y}\left(k+1\right)=CBu\left(k\right). \end{array}$$
2. Using the history of DBS inputs {*u*(1), *u*(2), ..., *u*(*k*)} to predict *y*(*k* + 1). Based on the fitted dynamic IO model 4, the predictor can be derived as the following forward recursion
  (10) $$\begin{array}{l} \{\begin{array}{l} \hat{x}\left(k+1\right)=A\hat{x}\left(k\right)+Bu\left(k\right),\\ \hat{y}\left(k+1\right)=C\hat{x}\left(k+1\right), \end{array} \end{array}$$
  with initial condition $\hat{x}\left(1\right)=0$.
3. Using the full history of both DBS inputs and output spectral activity {*u*(1), *u*(2), ..., *u*(*k*);*y*(1), *y*(2), .., *y*(*k*)} to predict *y*(*k* + 1). Based on the fitted dynamic IO model (4), the Kalman predictor can be written as the following:
  (11) $$\begin{array}{l} \{\begin{array}{l} \hat{x}\left(k\right)\;=A\hat{x}\left(k-1\right)+Bu\left(k-1\right)+L(y\left(k\right)\\ \;-C\left(A\hat{x}\left(k-1\right)+Bu\left(k-1\right)\right)),\\ {\hat{x}}_{p}\left(k+1\right)=A\hat{x}\left(k\right)+Bu\left(k\right),\\ \hat{y}\left(k+1\right)\;=C{\hat{x}}_{p}\left(k+1\right), \end{array} \end{array}$$
  with initial condition $\hat{x}\left(1\right)=0$.
4. We also evaluate a baseline prediction for comparison where we keep the output of training and test sets the same but randomly shuffle the time index of the input. We then use the history of the shuffled input and intact output to predict *y*(*k* + 1) using the Kalman predictor. Since the time indices of the input are manually shuffled in the modeling and mismatch with the time indices of the actual output, the prediction is essentially at random chance. The prediction error in this case thus provides an upper bound for the other three prediction methods above.

To quantify the offline system identification performance, we define the normalized prediction error (NPE) in the test set as


(12) $$\begin{array}{l} \text{NPE}=\sqrt{\frac{\sum_{k=0}^{J-1}{\left(y\left(k+1\right)-\hat{y}\left(k+1\right)\right)}^{2}}{\sum_{k=0}^{J-1}{\left(y\left(k+1\right)-\overset{-}{y}\right)}^{2}}}, \end{array}$$

where *J* = 1, 500 is the total time step in the test set, $\hat{y}$(*k* + 1) is the prediction using one of the methods above, $\overset{-}{y}=\frac{1}{J}\overset{J}{\underset{k=0}{\sum }}y\left(k+1\right)$ is time average. The denominator essentially uses the time average to predict *y*(*k* + 1). Thus, a useful dynamic IO model and prediction method should result in a NPE that is less than 1. We compare the NPEs of the four prediction methods across all 50 trials using the Wilcoxon signed-rank test.

#### 2.3.2. Online neuromodulation experiments

**Figure 3:**
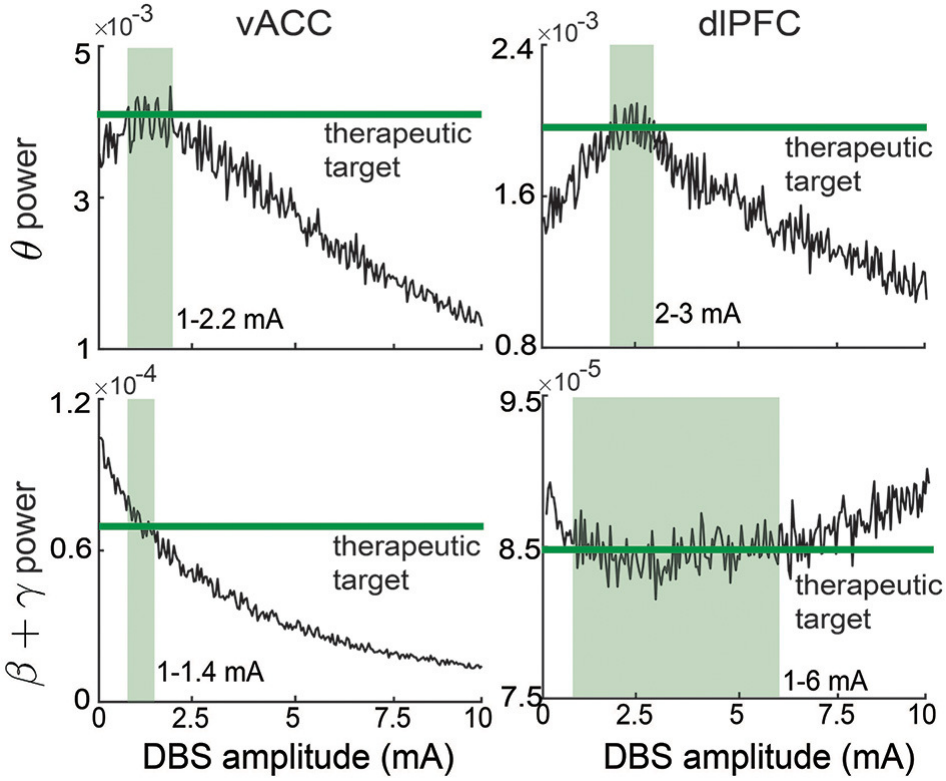
Therapeutic targets selection for vACC and dlPFC spectral activity. The therapeutic targets for each spectral activity are taken from the healthy state and shown in green. The light green region in each panel represents the corresponding ranges of DBS that can regulate the spectral activity to its corresponding target.

In online neuromodulation experiments, we test different neuromodulation methods in terms of their ability to regulate neural activity to track therapeutic targets. The therapeutic target is usually selected by the user before real-time BCI operation. The target variables should be selected as the neural features related to the MDD symptoms. Recent clinical findings have shown a close relationship between MDD symptoms and vACC-dlPFC spectral powers (Rao et al., 2018; Scangos et al., 2021a). Therefore, we select the vACC and dlPFC powers as the target variables, i.e., we aim to use the BCI to regulate *y*(*k*) to follow its target y^*^(*k*). We first run a set of experiments with constant therapeutic target values, i.e., y^*^(*k*) = y^*^, which does not change over time. The selection of the target value y^*^ should be related to the desired therapeutic effect. In our simulations, the target value of each element of y^*^ is taken as the corresponding vACC and dlPFC power value in the healthy state. The healthy vACC and dlPFC powers are computed by setting *I*_*dbs*_ = 0 and *f*_*D*_ = 1 in the vACC-dlPFC neural mass model (1) and then computing the spectral power as the same way in Equation (2). These therapeutic target values of each vACC and dlPFC power are shown as the constant green lines in Figure 3. A single trial of an online neuromodulation experiment lasts for *K* = 450 total time steps. DBS starts at time step 75 (*u*(*k*) = 0 for the first 75 time steps). We compare three neuromodulation methods.

1. Open-loop neuromodulation *u*^open^(*k*). Open-loop neuromodulation uses a fixed constant value for the DBS amplitude and does not change it over time, i.e.,
  (13) $$\begin{array}{l} u^{\text{open}}\left(k\right)=U. \end{array}$$
  In practice, the open-loop DBS amplitude *U* is determined by the clinician for each patient *via* a trial-and-error method. From Figure 3, we see that for our non-linear MDD brain, there is no single constant *U* that can simultaneously regulate the four output spectral powers to their individual target values. Therefore, without loss of generality, in each simulation trial, we choose *U* randomly from [1,6] mA that covers the range of amplitudes that can take at least one of the four output spectral powers to its target value. This range is also consistent with the typical open-loop DBS amplitude used for treating MDD in clinical applications (Rao et al., 2018).
2. Closed-loop responsive neuromodulation *u*^res^(*k*). Responsive neuromodulation works by first defining a scalar neural biomarker for mood symptoms and then triggering a constant DBS whenever the neural biomarker crosses a pre-defined threshold value. Responsive DBS is the state-of-the-art clinical closed-loop DBS treatment for MDD (Scangos et al., 2021a). This state-of-the-art responsive neuromodulation method uses the γ power of a single iEEG channel at a single limbic region as the scalar neural biomarker (Scangos et al., 2021a). Therefore, in our simulations, as an example, we choose to use the vACC β + γ power of the MDD brain as the neural biomarker in responsive neuromodulation. Accordingly, the pre-defined threshold value is set as the target value for vACC β + γ power. As a result, the responsive neuromodulation strategy is
  (14) $$\begin{array}{l} u^{\text{res}}\left(k\right)=\{\begin{array}{ll} 0, & y_{\beta +\gamma }^{v}\left(k\right)<y_{\beta +\gamma }^{v*},\\ U, & y_{\beta +\gamma }^{v}\left(k\right)\geq y_{\beta +\gamma }^{v*}. \end{array} \end{array}$$
  When DBS is triggered, its amplitude is set the same as in the open-loop case, which is the method used in current clinical implementation (Scangos et al., 2021a). It is worth noting that responsive DBS is not predictive because the threshold-crossing control strategy is not informed by a dynamic IO model and cannot predict the DBS effects on future spectral activity; thus, it is a sub-optimal, short-sighted control strategy.
3. Closed-loop predictive neuromodulation *u*^pred^(*k*). We apply Algorithm 1 in this case. Note that the predictive neuromodulation does not require a manual choice of a neural biomarker, the automatically estimated MDD brain state $\hat{x}\left(k\right)$ conceptually plays the role of a multi-dimensional neural biomarker.

In total, we run 100 trials of online neuromodulation experiments.

To further test our predictive neuromodulation methods, we run a more challenging set of online neuromodulation experiments. The simulation setup is the same as above except that the target values of vACC and dlPFC powers y^*^(*k*) are now allowed to change over time. Each target value is set as a stair-shape function (see Section 3.3 for the time-varying targets).

To quantify the online neuromodulation performance, we first average the controlled output trace at each time step across the 100 trials


(15) $$\begin{array}{l} \tilde{y}\left(k\right)=\frac{\sum_{i=1}^{N}y_{i}\left(k\right)}{N}, \end{array}$$

where *y*_*i*_(*k*) is the controlled output at time step *k* in trial *i* and *N* = 100 is the total number of trials. We then compute the normalized control error (NCE) over a fixed time window of length *K*_*c*_ = 10 time steps.


(16) $$\begin{array}{l} \text{NCE}=\sqrt{\frac{\sum_{k=t_{0}}^{t_{0}+K_{c}}{\left(\tilde{y}\left(k\right)-y^{*}\left(k\right)\right)}^{2}}{\sum_{k=t_{0}}^{t_{0}+K_{c}}y^{*}{\left(k\right)}^{2}}}, \end{array}$$

where *t*_0_ is the ending time step of the initial transition period after DBS turns on and is chosen as *t*_0_ = 100. We then slide the time window *K*_*c*_ without overlapping till the end of the online neuromodulation experiments, which gives us a total number of 35 realizations for NCE. We compare the NCE of the three neuromodulation methods across all 35 realizations using the Wilcoxon signed-rank test.

To quantify the battery consumption of different neuromodulation methods, we compute the input DBS energy (IE) similarly to the NCE:


(17) $$\begin{array}{l} \text{IE}=\frac{\sum_{k=t_{0}}^{t_{0}+K_{c}}\tilde{u}{\left(k\right)}^{2}}{K_{c}}. \end{array}$$

We similarly compare the IE of the three neuromodulation methods using the Wilcoxon signed-rank test.

#### 2.3.3. Simulations for personalized neuromodulation

Our primary goal is to use simulations to test our predictive neuromodulation method in a personalized neuromodulation framework. In this personalized framework, a given MDD subject is simulated by fixing *f*_*D*_ in the vACC-dlPFC neural mass model (1) because *f*_*D*_ is the key parameter in the model that determines MDD severity. The default value of *f*_*D*_ is fixed at 1.25, representing severe MDD. Then, we conduct dynamic system identification for this specific subject and fit a personalized linear state-space model (4). Next, we derive a personalized brain state estimator (7) and a personalized MPC (Equation 8) from the personalized linear state-space model. We then use the personalized brain state estimator and personalized MPC to form the personalized predictive neuromodulation system. We finally test the personalized predictive neuromodulation system in the same subject. Many previous closed-loop neuromodulation methods for PD and epilepsy have also been designed in a personalized neuromodulation framework (Santaniello et al., 2010; Liu et al., 2011; Ehrens et al., 2015; Bolus et al., 2018, 2021; Yang et al., 2018a; Su et al., 2019).

In our primary set of simulations, we use the personalized neuromodulation framework to test different neuromodulation methods within a single MDD subject with *f*_*D*_ = 1.25. Subsequently, if not explicitly mentioned, when we mention predictive neuromodulation, we mean the above personalized predictive neuromodulation. The results for this set of simulations are presented in Sections 3.1–3.3.

In an extended set of simulations, at the very beginning of the personalized neuromodulation framework, we vary the main MDD parameter *f*_*D*_ to simulate different subjects with different MDD severity. We then test if our predictive neuromodulation system can work in these subjects. In the original vACC-dlPFC neural mass model (Ramirez-Mahaluf et al., 2017), *f*_*D*_ = 1.25 represents severe MDD that needs DBS treatment and *f*_*D*_ = 1.15 represents moderate MDD where drug treatment may already be sufficient. Thus, we use *f*_*D*_ = 1.25 as the default MDD severity point and *f*_*D*_ = 1.15 as the lower limit for the MDD severity point that requires DBS treatment. Accordingly, we define the MDD severity deviation as


(18) $$\begin{array}{l} {\text{MDD}}_{\text{severity deviation}}=\;\frac{f_{D}-1.25}{1.25-1.15}\times 100\%, \end{array}$$

which quantifies the percentage deviation of a given subject from the default subject. In this set of simulations, we test 9 different subjects where we uniformly vary the MDD severity deviation from a large range [−100%, 100%] (corresponding to the *f*_*D*_ range [1.15, 1.35]). We test the personalized predictive neuromodulation system in each subject and evaluate the regulation performance using the normalized control error (NCE) in Equation (16). We then use linear correlation analyses to examine if the NCE of different subjects significantly changes as the MDD severity deviation changes.

To gain insights into how the fitted personalized model affects the regulation performance. We investigate two critical control-theoretic properties of the fitted personalized model, i.e., controllability and observability. Specifically, from a control-theoretic perspective, the effectiveness of the estimator usually depends on the observability of the fitted model, and the effectiveness of the controller usually depends on the controllability of the fitted model (Wang et al., 2017). We compute the controllability matrix of the fitted linear state space model (4) as


(19) $$\begin{array}{l} M_{C}=\left[A,\;AB,\;A^{2}B,\;\ldots ,\;A^{n_{x}-1}B\right], \end{array}$$

and quantify the controllability condition of the fitted model as the inverse condition number of *M*_*C*_ (Aguirre et al., 2018):


(20) $$\begin{array}{l} \kappa \left(M_{C}\right)=\frac{\sigma_{\text{min}}\left(M_{C}\right)}{\sigma_{\text{max}}\left(M_{C}\right)}, \end{array}$$

where σ_max_ (·) and σ_min_ (·) are the maximum and minimum singular values of a given matrix. A larger κ(*M*_*C*_) corresponds to a better controllability condition, i.e., the model requires less energy to realize precise control (Wang et al., 2017). Similarly, we compute the observability matrix of the fitted linear state space model (4) as


(21) $$\begin{array}{l} M_{O}={\left[C,\;CA,\;CA^{2},\;\ldots ,\;CA^{n_{x}-1}\right]}^{\prime }, \end{array}$$

and quantify the observability condition of the fitted model as the inverse condition number of *M*_*O*_ (Aguirre et al., 2018):


(22) $$\begin{array}{l} \kappa \left(M_{O}\right)=\frac{\sigma_{\text{min}}\left(M_{O}\right)}{\sigma_{\text{max}}\left(M_{O}\right)}. \end{array}$$

Similarly, a larger κ(*M*_*O*_) corresponds to a better observability condition, i.e., the brain state estimator requires fewer past neural activity to estimate the present brain state (Aguirre et al., 2018). Therefore, we compute the κ(*M*_*C*_) and κ(*M*_*O*_) for each personalized model. We next use linear correlation analyses to investigate if κ(*M*_*C*_) and κ(*M*_*O*_) significantly correlate with the MDD severity deviation across subjects. The results for the extended simulations and controllability/observability analyses are presented in Section 3.4.

#### 2.3.4. Simulations for nominal neuromodulation

To investigate the robustness of the neuromodulation system, we test our predictive neuromodulation method in a more challenging scenario, i.e., a nominal neuromodulation framework. In this framework, a nominal MDD subject is first simulated by fixing *f*_*D*_ = 1.25 in the vACC-dlPFC neural mass model (Equation 1). Then, we conduct dynamic system identification for this nominal subject and fit a nominal linear state-space model (Equation 4). Next, we derive a nominal brain state estimator (Equation 7) and a nominal MPC (Equation 8) from the identified nominal linear state-space model. Together, we combine the nominal brain state estimator and nominal MPC to build the nominal predictive neuromodulation system. We finally test the nominal predictive neuromodulation system in new subjects with different MDD severity. Different test subjects are simulated by only varying the key MDD parameter *f*_*D*_ while keeping the rest vACC-dlPFC model parameters unchanged. Although any of the vACC-dlPFC model parameters can in principle vary from one subject to another, our simulations here focus on the evaluation of regulation performance against one of the key parameters, i.e., *f*_*D*_ that quantifies different MDD severity. Note that nominal neuromodulation is conceptually more challenging than personalized neuromodulation because the same nominal neuromodulation system needs to be robust enough to achieve regulation for different test subjects. Such a nominal neuromodulation framework is usually used to develop and test robust neural controllers (Westover et al., 2015).

There, we use the nominal neuromodulation framework to test the robustness of our predictive neuromodulation method. We test 9 different subjects where we uniformly vary the MDD severity deviation from the range [−20%, 20%]. We apply the nominal predictive neuromodulation system in each test subject and evaluate the regulation performance using NCE in Equation (16). We then use linear correlation analyses to examine if the NCE of different test subjects significantly changes as the MDD severity deviation changes. We then examine how the NCE of different test subjects changes as the MDD severity deviation changes. We also evaluate the MDD severity deviation boundary where the nominal predictive neuromodulation system performs no better than the open-loop neuromodulation system. The results for this set of nominal neuromodulation simulations are presented in Section 3.5.

## 3. Results

### 3.1. Dynamic system identification enabled the prediction of vACC-dlPFC multiband spectral activity

**Figure 4:**
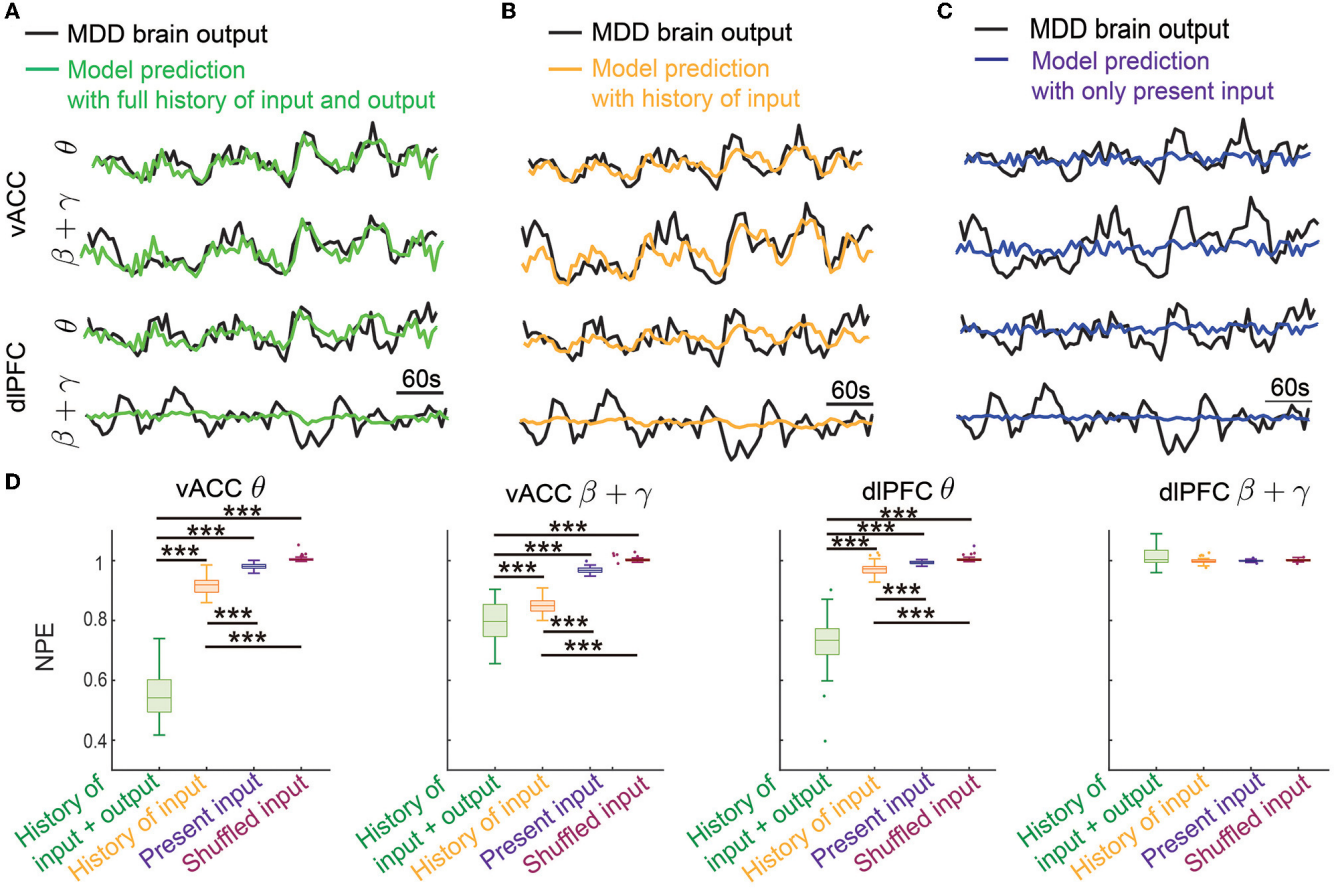
Dynamic system identification enabled the prediction of vACC-dlPFC multiband spectral activity. **(A)** The prediction of vACC-dlPFC multiband spectral activity using the full history of input and output data. **(B)** The prediction of vACC-dlPFC multiband spectral activity using the history of only input data. **(C)** The prediction of vACC-dlPFC multiband spectral activity using only the present input. **(D)** Comparisons of prediction error. In the boxplot, the middle line inside the box shows the median, box edges show the 25th and 75th percentiles, whiskers represent the minimum and maximum values and dots show outlier values. Outliers are the points that are more than 1.5 times the interquartile distance, i.e., the box size, away from the end and beginning of the box. Asterisks indicate significance (***P < 0.0001).

In offline dynamic system identification experiments, we trained and tested the dynamic IO models in terms of predicting vACC-dlPFC multiband spectral activity. Figure 4A shows the results in one example test set. In this case, we implemented the Kalman predictor derived from the trained dynamic IO model, which predicted the future spectral activity from its own past (history of output) and the past DBS pattern (history of input). We achieved good prediction for vACC θ power, vACC β + γ power, and dlPFC θ power, but not as good for dlPFC β + γ power (see Section 4). The ability to predict future spectral activity using the history of both input and output data was key in allowing the dynamic brain state estimator to accurately estimate the MDD brain state in subsequent online neuromodulation.

To show that the IO model is indeed using the dynamic information in spectral activity for achieving good prediction, we gradually removed the history information from the prediction. We first removed the history of the output data and only kept the history of input for prediction. The prediction performance qualitatively became worse in this case (Figure 4B) but still tracked the general trend of the dynamics. The ability of only using the history of input data for prediction allowed the MPC to predict the DBS effects on future spectral activity, which subsequently led to accurate control online neuromodulation (see Sections 3.2 and 3.3). We next further removed the history of the input data and only kept the present input for prediction. This was essentially a static prediction not informed by the dynamics of past input and output. In this case, the prediction became much worse and did not track the spectral activity dynamics (Figure 4C).

We quantified the above observations in 50 trials of independent training and testing. We found that the prediction error using the full history of input and output was significantly smaller than the other two prediction errors for the vACC θ power, vACC β + γ power, and dlPFC θ power (first three panels in Figure 4A, Wilcoxon signed-rank test *P* < 10^−10^ for all comparisons). The prediction error was also significantly smaller than the baseline prediction error using shuffled input, showing that our dynamic IO model was not overfitted to training data (first three panels in Figure 4A, Wilcoxon signed-rank test *P* < 10^−10^ for all comparisons). Notably, when only keeping the history of input data for prediction, the prediction error was still significantly smaller than the static prediction error and the baseline prediction error (first three panels in Figure 4A, Wilcoxon signed-rank test *P* < 10^−10^ for all comparisons). By contrast, the static prediction error stayed close to the baseline prediction error. These results showed that our dynamic IO model indeed captured the dynamics in the spectral activity and enabled the prediction of future spectral activity using the history of output and input data or using the history of only input data. However, we noted that in all cases, the prediction for the dlPFC β + γ band power (the last panel in Figure 4D) was not different from the baseline prediction, indicating that the dynamics in dlPFC β + γ band power probably exhibited complex non-linear dynamics not captured by our linear dynamic IO model (see Section 4).

### 3.2. Predictive neuromodulation accurately regulated the vACC-dlPFC multiband spectral activity in an energy-efficient manner

**Figure 5:**
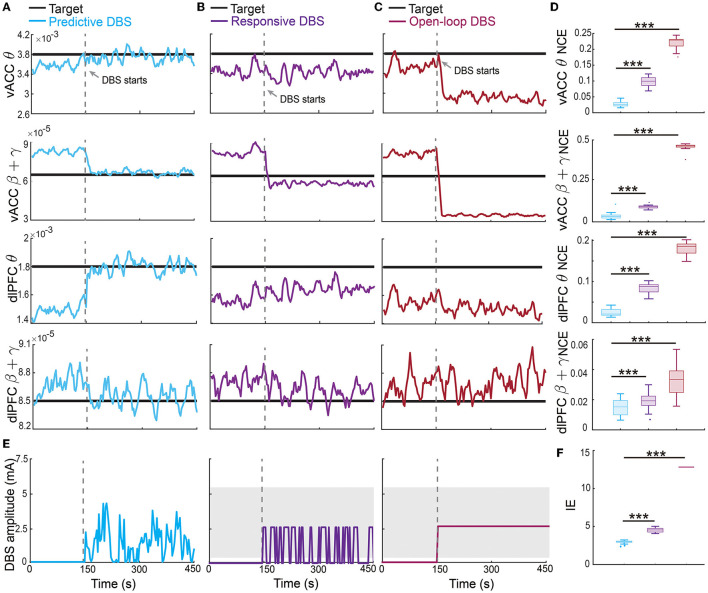
Predictive neuromodulation accurately regulated the vACC-dlPFC multiband spectral activity in an energy-efficient manner. **(A)** Online neuromodulation results using predictive DBS. Each row represents one spectral activity. The solid colored line represents the controlled spectral activity averaged over 100 trials. The thick dark line represents the therapeutic target. The vertical dashed line represents the time when DBS starts. **(B)** Same as **(A)** but for responsive DBS. **(C)** Same as **(A)** but for open-loop DBS. **(D)** Comparisons of control error for different online neuromodulation methods. Cyan represents predictive DBS; purple represents responsive DBS; dark red represents open-loop DBS. Each row represents one spectral activity. The boxplot styles are the same as Figure 4D. **(E)** Example single-trial trace of controlled DBS amplitude for the predictive method (left, cyan), responsive method (middle, purple), and open-loop method (right, dark red). The gray shaded area is the DBS amplitude range where the constant input in open-loop and responsive DBS is chosen from. Specifically, in each simulation trial, one single constant value *U* is first chosen from the gray range and then fixed during the neuromodulation process to implement the open-loop and responsive DBS as in Equations (13) and (14). **(F)** Comparison of input DBS energy for the predictive method (cyan), responsive method (purple), and open-loop method (dark red). The boxplot styles are the same as Figure 4D. Asterisks indicate significance (***P < 0.0001).

The offline fitted dynamic IO model enabled us to design an online predictive neuromodulation system to regulate vACC-dlPFC multiband spectral activity in MDD. In this set of online experiments, we used constant therapeutic targets for each spectral activity, which were set as the spectral powers of the healthy state (see Figure 3). Figure 5A shows the controlled spectral activities averaged across 100 independent trials of online neuromodulation. Each row represents the control result for one spectral power activity. Taking the vACC β + γ power as an example (the second row in Figure 5A), before DBS starts, the vACC β + γ power in the MDD state was far away from the therapeutic target. Once predictive DBS turns on, the system took all four spectral activities as feedback and adjusted the DBS amplitude accordingly in real time (Figure 5E, left panel). The predictive DBS successfully regulated the vACC β + γ activity to track the therapeutic target at the steady state, achieving a small control error (NCE was 0.0315 [0.0233, 0.0417], mean and 95% confidence interval, see the second row in Figure 5D). Similar results held for the other three spectral activities.

By contrast, the state-of-the-art responsive DBS used a simple threshold-crossing control strategy that cannot predict the spectral activity dynamics (Figure 5E, middle panel). Therefore, while the vACC β + γ power was reduced by responsive DBS toward the target, it decreased too much to be under the desired target (second row in Figure 5B), which could translate to over-treatment and side effects in clinical practice. Thus, responsive DBS did not accurately regulate the spectral activities, resulting in a larger control error that was almost three times of the predictive DBS (responsive DBS NCE 0.0919 [0.0850, 0.0974] v.s. predictive DBS NCE 0.0315 [0.0233, 0.0417], Wilcoxon signed-rank test *P* < 10^−10^, see the second row in Figure 5D). Similar results held for the other three spectral activities.

The open-loop DBS performed the worst among the three neuromodulation methods because it used a constant DBS amplitude without the guidance of the real-time spectral activities (Figure 5E, right panel). As a result, open-loop DBS resulted in more severe over-treatment of the vACC β + γ power (second row in Figure 5C); it thus had the largest control error, which was more than 15 times of predictive DBS (open-loop DBS NCE 0.4633 [0.4651, 0.4680] vs. predictive DBS NCE 0.0315 [0.0233, 0.0417], Wilcoxon signed-rank test *P* < 10^−10^, see the second row in Figure 5D). Similar results held for the other three spectral activities. Interestingly, we found that the predictive DBS for dlPFC β + γ power had a relatively large control variance and was not as good as the other three spectral activities. This was likely because the dynamic IO model did not predict dlPFC β + γ as well (the last panel in Figure 4D), hindering the optimal control of dlPFC β + γ. Nevertheless, the control performance of responsive DBS for dlPFC β + γ still outperformed responsive and open-loop DBS (the fourth row in Figure 5), suggesting the advantage of joint feedback control of all four spectral activity.

**Figure 6:**
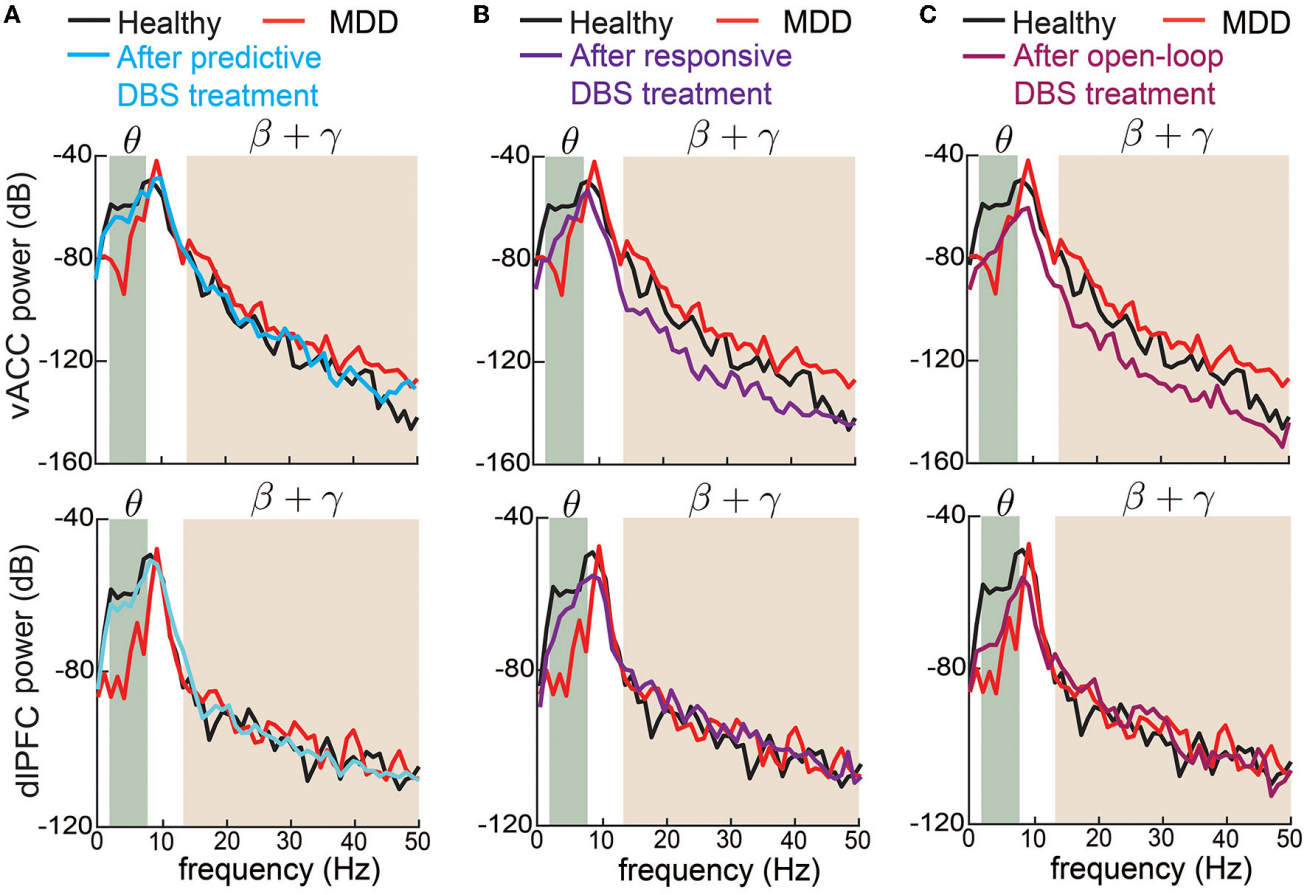
Changes in vACC and dlPFC power spectrum before and after DBS treatments. **(A)** Changes in vACC (top) and dlPFC (bottom) power spectrum before (red) and after (cyan) predictive DBS treatments. The power spectrum in the healthy state is shown in black. The shaded area represents the θ and β + γ bands that are of interest. **(B)** Same as **(A)** but for responsive DBS. **(C)** Same as **(A)** but for open-loop DBS.

We also took a closer look at the typical vACC and dlPFC power spectrum before and after DBS treatments (Figure 6). Taking the vACC power spectrum as an example (first row in Figure 6), we see that before treatment, the MDD θ power was below the healthy spectrum and the MDD β + γ power was above the healthy spectrum. After predictive DBS treatment (Figure 6A), the θ power was elevated, and the β + γ power was suppressed. Together, the spectrum after predictive DBS treatment stayed close to the healthy spectrum, indicating optimal treatment. The change in the β + γ power appeared small because we used the conventional dB scale for the vertical axis; readers can refer to Supplementary Figure 1 for a more visible comparison of the β + γ power spectrum in linear scale. By contrast, while the responsive DBS elevated the θ power, it did not elevate it enough to match the healthy spectrum (possible under-treatment), and the β + γ power was suppressed too much to be under the healthy spectrum (possible over-treatment, Figure 6B). Open-loop DBS resulted in even worse control performance (Figure 6C). Similar results held for the dlPFC power spectrum.

**Figure 7:**
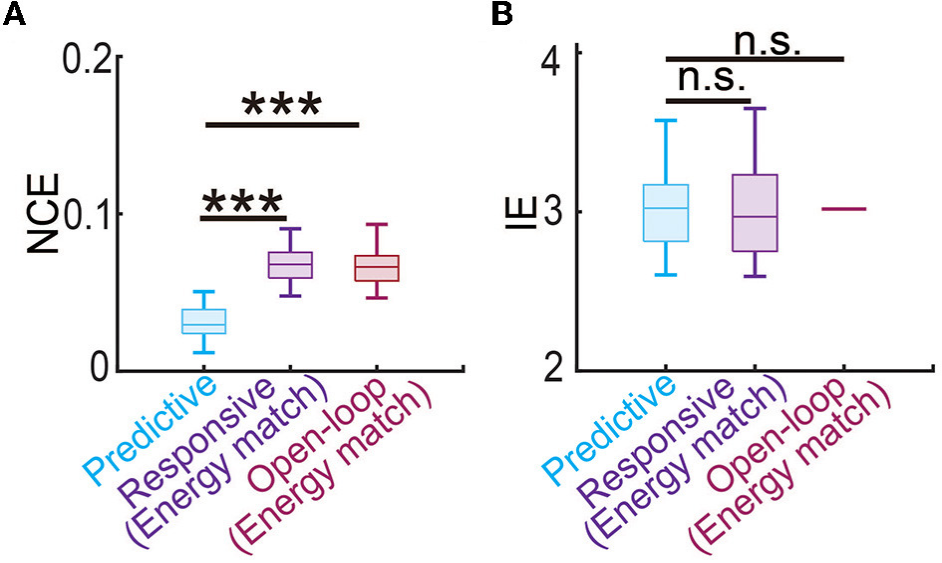
Predictive neuromodulation maintained the best regulation performance even when using a similar level of input energy. **(A)** The normalized control error in the energy-matched case. The boxplot styles are the same as in Figure 4D. **(B)** The input energy in the energy-matched case. The boxplot styles are the same as in Figure 4D. *n*.*s*., *P* > 0.05. Asterisks indicate significance (***P < 0.0001).

Finally, we estimated the battery power consumption of each neuromodulation method by computing the energy of the DBS input (Figure 5F). We found that predictive DBS resulted in the smallest DBS energy compared with responsive and open-loop DBS (Wilcoxon signed-rank test *P* < 10^−10^). We also did another analysis where instead of randomly choosing the stimulation amplitude *U* in open-loop and responsive DBS, we manually chose *U* to be the mean value of the stimulation amplitude of predictive DBS. We then re-implemented open-loop and responsive DBS with this new choice of *U*. We found that by doing so, the energy of the open-loop, responsive and predictive DBS was not significantly different from each other (Wilcoxon signed-rank test, *P*>0.3130, Figure 7B). However, even in this energy-matched case, the regulation performance of predictive DBS was significantly better than the open-loop and responsive DBS (Wilcoxon signed-rank test, *P* < 10^−10^, Figure 7A). To summarize, the predictive neuromodulation method accurately regulated two-region multiband spectral activity to therapeutic targets, achieving significantly smaller control error and DBS energy than state-of-the-art open-loop and responsive neuromodulation methods.

### 3.3. Predictive neuromodulation generalized accurate regulation performance across time-varying therapeutic targets

**Figure 8:**
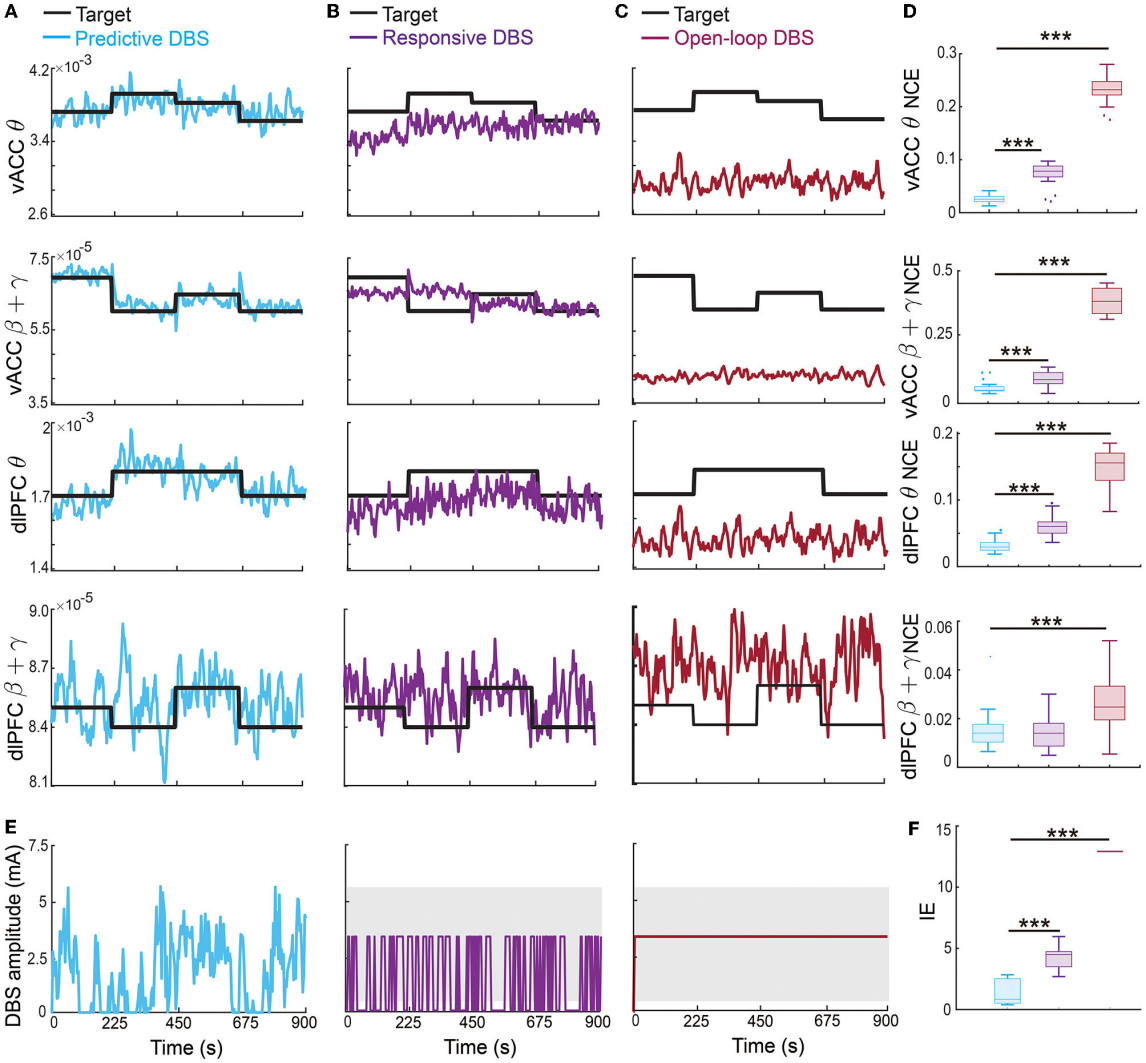
Predictive neuromodulation generalized accurate regulation performance across time-varying therapeutic targets. Figure legends are the same as Figure 5, except that the therapeutic targets were time-varying in this case [thick black lines in **(A–C)**]. Asterisks indicate significance (***P < 0.0001).

The therapeutic targets may need to change over time depending on the patient's clinical needs. Therefore, in this set of online experiments, we investigated the more challenging problem of regulating the vACC-dlPFC multiband spectral activity to follow time-varying therapeutic targets. We found that the predictive neuromodulation BCI system generalized accurate regulation performance across time-varying therapeutic targets for vACC θ, vACC β + γ and dlPFC θ (Figure 8). Again taking the vACC β + γ power as an example (second row in Figure 8), the predictive DBS accurately regulated vACC β + γ power to track the stair-shape time-varying therapeutic targets (the second row in Figure 8A, NCE 0.0269 [0.0245, 0.0413]).

The responsive DBS regulated vACC β + γ power to stay around the time-varying therapeutic targets because it nevertheless used real-time spectral activities as feedback, but cannot achieve accurate regulation, resulting in control errors that were more than twice of predictive DBS (the second row in Figures 8B, D, responsive DBS NCE 0.0687 [0.0533, 0.0959] v.s. predictive DBS NCE 0.0269 [0.0245, 0.0413], Wilcoxon signed-rank test *P* < 10^−10^). Open-loop DBS did not even regulate vACC β + γ power to stay around the time-varying therapeutic targets because it did not use real-time spectral activities as feedback, resulting in control errors that were more than 10 times of predictive DBS (the second row in Figures 8C, D, open-loop DBS NCE 0.3725 [0.3258, 0.4242] v.s. predictive DBS NCE 0.0269 [0.0245, 0.0413], Wilcoxon signed-rank test *P* < 10^−10^). Similar results held for vACC θ power and dlPFC θ power. We note that dlPFC β + γ power had a larger control variance and was difficult to regulate to follow time-varying therapeutic targets.

Moreover, predictive DBS led to a significantly smaller input DBS energy than the other two methods (Figures 8E, F, Wilcoxon signed-rank test *P* < 10^−10^). The above results show that predictive DBS can generalize the accurate regulation performance across multiple time-varying therapeutic targets and significantly outperformed responsive DBS and open-loop DBS.

### 3.4. Personalized predictive neuromodulation maintained accurate regulation performance in subjects with different MDD severity

**Figure 9:**
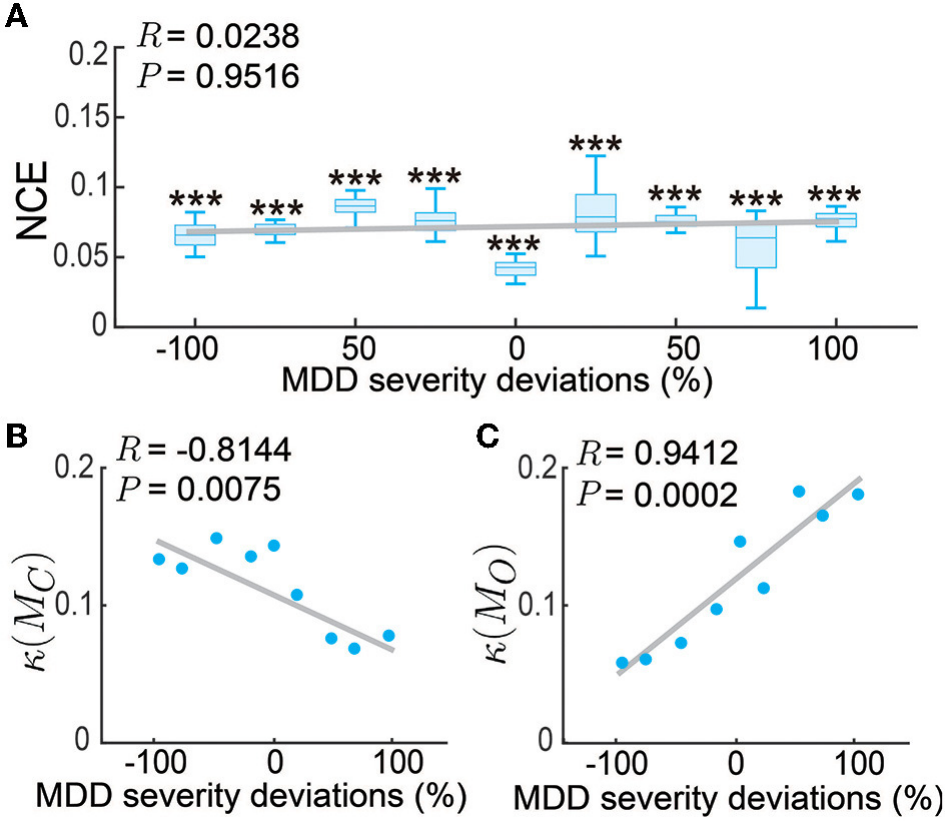
Personalized predictive neuromodulation maintained accurate regulation performance in subjects with different MDD severity. **(A)** The normalized control error (NCE) across subjects with different MDD severity deviations from the default subject. The x-axis is the MDD severity deviation, where 0% represents the default subject with *f*_*D*_ = 1.25. The boxplot styles are the same as in Figure 4D. ^***^P < 0.0005 when compared with open-loop neuromodulation in the same subject. The gray line is the linear fit of the medians in each subject using Pearson's linear correlation analysis. The correlation coefficient *R* and Pearson's *P*-value are indicated in the upper left corner. **(B)** The controllability condition of the personalized model across subjects. Figure conventions are the same as **(A)**. **(C)** The observability condition of the personalized model across subjects. Figure conventions are the same as **(A)**.

Up to now, we have tested our personalized predictive neuromodulation system on a single default subject whose MDD severity parameter was set as *f*_*D*_ = 1.25. We further tested the personalized predictive neuromodulation system in subjects with different MDD severity (see Section 2.3.3). We tested 9 subjects whose MDD severity deviation from the default subject had a large range from −100% to 100%. We found that the personalized predictive neuromodulation system maintained accurate regulation performance in each subject. In each of the 9 subjects, the normalized control error of personalized predictive neuromodulation remained significantly smaller than open-loop neuromodulation (Wilcoxon signed rank test *P* < 10^−10^ for all comparisons, Figure 9A). Further, linear correlation analyses showed that the NCE did not significantly change with the MDD severity deviation (Pearson's correlation coefficient *R* = 0.0238, *P* = 0.9516). This result showed that personalized predictive neuromodulation successfully maintained accurate regulation performance in subjects with a wide range of MDD severity.

To gain insights into why the personalized predictive neuromodulation maintained accurate regulation performance, we next investigated the controllability and observability properties of the fitted personalized models (see Section 2.3.3). We found that the inverse condition number of the personalized model's controllability matrix, i.e., κ(*M*_*C*_) in Equation (21), was negatively correlated with the subjects' MDD severity (Figure 9B). This result indicated that as the MDD severity increased, the controllability condition worsened, suggesting that the models became harder to control. We also found that the inverse condition number of the personalized model's observability matrix, i.e., κ(*M*_*O*_) in Equation (22), was positively correlated with the subjects' MDD severity (Figure 9C). This result indicates that as the MDD severity increased, the observability condition improved, suggesting that the underlying MDD brain state of the model was easier to estimate. The estimator and controller worked jointly to form the entire predictive neuromodulation system. Thus, as the MDD severity increased, the worse controllability condition and the better observability condition might counteract each other, keeping the overall regulation performance at a relatively constant level (Figure 9A). The investigation of the detailed mechanism of such counteraction is beyond the scope of this paper and is an interesting future research direction.

### 3.5. Investigating the robustness of the nominal predictive neuromodulation system

**Figure 10:**
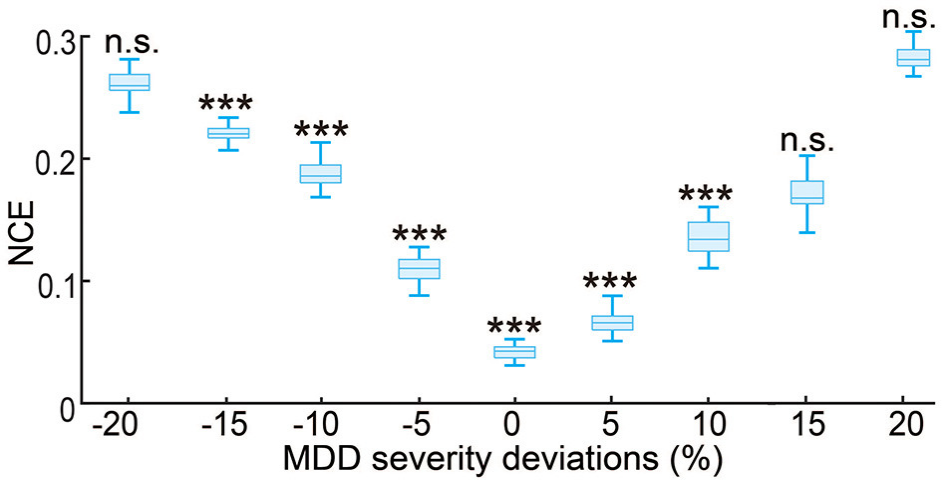
Robustness of the nominal predictive neuromodulation system. The x-axis is the MDD severity deviation for different test subjects, where 0% represents the nominal MDD severity of *f*_*D*_ = 1.25. The boxplot styles are the same as in Figure 4D. ^***^P < 0.0005 when compared with open-loop neuromodulation. *n*.*s*.: *P* > 0.5 when compared with open-loop neuromodulation.

The results so far have evaluated the performance of the predictive neuromodulation system in a personalized neuromodulation framework, where neuromodulation system design and testing were conducted in the same simulated subject. In this section, we further investigated the robustness of the predictive neuromodulation system, where a nominal system was designed in a nominal MDD subject and then tested in subjects with different MDD severity (see Section 2.3.4). As expected, as the test subject's MDD severity deviated more from the nominal subject, the control error for the nominal predictive neuromodulation system increased (Figure 10). However, within the MDD deviation range of [−15%, 10%], the nominal predictive neuromodulation system still significantly outperformed the open-loop neuromodulation (Wilcoxon signed rank test *P* < 0.0005 for all comparisons). This result suggests that the nominal predictive neuromodulation system is robust to the change in subject MDD severity to a reasonable extent. On the other hand, we also noticed that in test subjects whose MDD severity was very different from the nominal value (when MDD deviation was less than −20% or more than 15%), the nominal predictive neuromodulation performed no better than the open-loop neuromodulation, indicating that the nominal model fitted in offline system identification might not adequately work for a wide range of test subjects (see Section 4).

## 4. Discussion

Over the past decade, there has been much work in developing model-based closed-loop neuromodulation methods for neurological and neuropsychiatric disorders (Santaniello et al., 2010; Liu et al., 2011; Ehrens et al., 2015; Nagaraj et al., 2017; Bolus et al., 2018, 2021; Yang et al., 2018a, 2021b; Su et al., 2019; Fang and Yang, 2021, 2022; Zhu et al., 2021). These studies share a typical framework of first conducting offline system identification to fit a model, then designing feedback controllers based on the fitted model, and finally using the controller for online closed-loop neuromodulation. We also adopted the same framework in this work. However, prior studies have mainly focused on neurological disorders such as Parkinson's disease (PD) (Santaniello et al., 2010; Liu et al., 2011; Su et al., 2019; Zhu et al., 2021) and epilepsy (Ehrens et al., 2015; Nagaraj et al., 2017) but not neuropsychiatric disorders such as MDD. This is likely because a good understanding of the disease mechanism of MDD is still lacking (Mayberg, 1997; Drevets, 2001; Williams, 2017). Recent clinical studies of mood symptoms have suggested that neural activity from multiple sites and multiple frequency bands can be related to mood (Kirkby et al., 2018; Rao et al., 2018; Sani et al., 2018; Smart et al., 2018; Scangos et al., 2021a; Bijanzadeh et al., 2022; Smith et al., 2022; Xiao et al., 2023). By contrast, model-based DBS methods for PD and epilepsy usually assume that the neural activity comes from a local brain region—e.g., basal ganglia in PD (Santaniello et al., 2010; Liu et al., 2011; Su et al., 2019; Zhu et al., 2021)—or a single frequency band—e.g., β band in PD (Su et al., 2019; Zhu et al., 2021). Our prior work proposes an extension to multi-input multi-output regulation of brain states (Yang et al., 2018a, 2021b) but was tested in a general dynamic model that is not tightly connected with MDD. The study here is unique in the sense that we developed a BCI system of predictive neuromodulation that regulates the multiband neural activity for MDD, and specifically showed the accurate control performance of the system in a biophysically plausible vACC-dlPFC neural mass model of MDD.

Model-based closed-loop neuromodulation methods for PD and epilepsy are usually tested in simulations that involve some model of the disease under consideration (Santaniello et al., 2010; Liu et al., 2011; Ehrens et al., 2015; Nagaraj et al., 2017; Su et al., 2019; Zhu et al., 2021). Using simulation validation is a common practice in developing new neurotechnologies before moving to clinical experiments (Cunningham et al., 2011; Charles et al., 2014; Citi et al., 2014; Shapero et al., 2014; Greco et al., 2015; Yang and Shanechi, 2016; Bolus et al., 2018; Yang et al., 2018a, 2019a, 2021a; Wang et al., 2021; Xu and Wang, 2021). Given MDD is a complex network-level disorder (Mayberg, 1997; Drevets, 2001; Williams, 2017), as the prerequisite for clinical implementation, we also tested our system in a biophysically plausible model of MDD. We chose to use a well-known vACC-dlPFC neural mass model of MDD (Ramirez-Mahaluf et al., 2017) because it models the interaction between key limbic and frontal regions (Mayberg, 1997; Fox et al., 2012). More importantly, the vACC-dlPFC neural mass model exhibits complex non-linear dynamics such as Hopf bifurcation and limit cycle that are biophysically meaningful for MDD (Ramirez-Mahaluf et al., 2017). However, there still exists a gap between the model in its original form and clinical findings because the original model focuses on the firing rate activity dynamics while clinical practice focuses more on spectral activity dynamics. We thus made several adjustments to the original model to construct a MDD brain model that takes DBS amplitude as input and generates multiband spectral activity as the output. The vACC-dlPFC neural mass model served as a simulation testbed to test our dynamic system identification and predictive neuromodulation methods. Within this testbed, we successfully showed that predictive neuromodulation outperformed open-loop and responsive neuromodulation for MDD. Our results also demonstrated interesting non-linear responses and temporal dynamics in the simulated vACC-dlPFC multiband spectral activity. Therefore, using such a model as the simulation tested can take the BCI system of predictive neuromodulation a step further toward clinical testing.

However, the vACC-dlPFC neural mass model for MDD has its limitations: it only models a small two-region subnetwork of a much larger limbic-frontal network that has been shown to regulate mood (Williams, 2017); it only models spectral powers as the output signal while more complex network interactions such as spectral coherence can also be related to mood (Kirkby et al., 2018); it is a mesoscopic model that models the aggregated activity of neural masses, which ignores microscopic single neuron spiking dynamics that are related to mood states (Yang et al., 2018b). With the advance of the research into the mechanism of MDD, more precise and detailed computational models can be used to test our predictive neuromodulation system further. Finally, regardless of the biophysical model being used, simulations only provide the first step in developing neuromodulation systems. The ultimate validation of neuromodulation systems requires carefully designed clinical experiments and is a critical topic for future investigation.

Despite the above limitations, the vACC-dlPFC model has indeed revealed important non-linear neural dynamics underlying MDD (Ramirez-Mahaluf et al., 2017). While the neural dynamics are non-linear, from a control-theoretic perspective, linear estimators and controllers have several advantages in building useful BCI systems for treating MDD. First, the aggregated mesoscopic neural dynamics originating from non-linear microscopic non-linear neural activity can show important linear characteristics. For example, Linear dynamic models have been shown to be a powerful model to track mesoscopic and macroscopic neural dynamics such as those seen in population firing rates (Petreska et al., 2011; Aghagolzadeh and Truccolo, 2015; Kao et al., 2015; Sani et al., 2021), local field potential (Yang et al., 2021b), and iEEG (Yang et al., 2019b). Second, a better approximation of non-linear neural dynamics can be achieved by increasing the linear model orders, such as the state dimension in linear state space models (Yang et al., 2019b). Third, directly using non-linear models to design estimators and controllers may lead to instability and increased sensitivity to noise (Bertsekas, 2012; Camacho and Alba, 2013; Fang and Yang, 2022). Thus, linear models are amenable to designing stable and robust estimators and controllers, leading to their frequent use in designing closed-loop neuromodulation in PD and epilepsy (Ehrens et al., 2015; Su et al., 2019). Due to the above reasons, we chose to identify a simplified linear dynamic IO model to capture these spectral activity dynamics. In our simulations, we successfully used the dynamic linear state-space model to predict the non-linear dynamics in the vACC-dlPFC neural mass model and subsequently used the model to design the dynamic brain state estimator and MPC. We found that the linear dynamic model enabled accurate offline prediction and online control for three of the spectral activities, significantly outperforming the state-of-the-art responsive DBS. This can be explained by two reasons. First, responsive DBS cannot predict future brain activity to make stimulation adjustments accordingly. Second, responsive DBS uses a simple threshold-crossing control strategy designed in an *ad-hoc* manner without adopting optimal control methods. Thus, responsive DBS did not precisely regulate the neural activity to its target values. On the other hand, we found that our methods performed worse for the dlPFC β + γ power. This is likely because the dlPFC β + γ power involves complex non-linear dynamics that are not captured by the linear model. Therefore, one future direction is develop non-linear and adaptive IO models (Ahmadipour et al., 2021; Fang and Yang, 2021, 2022; Yang et al., 2021a) to help improve the prediction and control of non-linear spectral activity dynamics in MDD.

We tested two ways of implementing our predictive neuromodulation system, i.e., the personalized and nominal frameworks. The personalized framework fits a personalized model for each subject, and we found the corresponding predictive neuromodulation system maintained accurate control performance in subjects with a large range of MDD severity differences. Fitting a personalized model for each subject may increase the burden of data collection in practice and interrupt treatment (Westover et al., 2015; Yang and Shanechi, 2016). By contrast, the nominal framework uses a single nominal model for all subjects, which removes the need to fit a different model for each subject but at the cost of reduced robustness; it can only maintain satisfactory control performance within a limited range of MDD severity deviations. Indeed, a single nominal model may not be sufficient to describe all the complex neural dynamics across a large number of subjects since different subjects have different neuronal physiology and connectivity (Ferrat et al., 2018). One promising way of combining the benefits of the two frameworks is to develop adaptive neuromodulation systems that can automatically use personalized data to adapt the nominal model during the neuromodulation process (Yang et al., 2019a; Zhu et al., 2021; Fang and Yang, 2022), which is an important future research direction.

The future clinical implementation and application of our predictive neuromodulation system require solving several critical challenges. First, selecting the most effective network neural features that are related to MDD symptoms is still an open problem. Recent studies have indicated that some mood-related neural features can be common across patients, but some can be personalized (Kirkby et al., 2018; Sani et al., 2018; Bijanzadeh et al., 2022; Xiao et al., 2023). Future work can use the proposed multiband modeling framework for aggregating different types of neural features for the best estimate of MDD brain states. Second, based on the personalized mood-related neural features, the corresponding therapeutic targets can also be personalized in different patients. For example, we may identify the range of mood-related neural features that correspond to relieved symptoms for a given patient. Clinicians can then set the therapeutic target to be within this range. Third, building a dynamic IO model for predicting multiband neural responses to DBS in MDD patients is key in enabling predictive control. Our prior work has built such models in non-human-primate experiments (Yang et al., 2021b). Extending and testing such models for humans and especially for MDD patients is a critical future direction (Shanechi, 2019). Fourth, in online neuromodulation experiments, many factors—such as neural plasticity, patient movements, and environmental noise—can lead to real-time unknown disturbances that can severely degrade DBS control performance or even lead to instability. Thus the predictive neuromodulation BCI system should incorporate robust and adaptive components to address noise, disturbance, and non-stationarity in the neural activity dynamics (Bolus et al., 2021; Fang and Yang, 2021, 2022; Zhu et al., 2021), which is another important future direction. Finally, in terms of the clinical criteria for evaluating neuromodulation BCI systems, the criteria for current open-loop DBS are usually based on clinical questionnaire scores such as the Montgomery-Åsberg Depression Rating Scale (MADRS) (Mayberg et al., 2005; Holtzheimer et al., 2017) and the Hamilton Depression Rating Scale (HDRS) (Mayberg et al., 2005; Holtzheimer et al., 2017). However, the registration of these scales takes time and cannot be obtained continuously over time. BCI systems can identify neural biomarkers of mood symptoms and monitor the neural biomarkers continuously over time. Thus, the quantitative changes in the neural biomarkers can provide another criterion that can supplement the clinical questionnaire scores (Shanechi, 2019). The dynamic brain state estimator in our framework provides a framework for identifying such neural biomarkers. In our simulations, we used the changes in mood-related neural features to quantify the treatment effect. Future work should investigate if the combined changes in neural biomarkers and clinical questionnaire scores can better reflect the treatment outcomes.

## 5. Conclusion

With the goal of improving current open-loop and responsive neuromodulation treatments for MDD, we develop a new BCI system of predictive neuromodulation. We then comprehensively test the system using a simulation testbed that incorporates a biophysically plausible vACC-dlPFC neural mass model of MDD. Our results show that the proposed predictive neuromodulation system can accurately predict the non-linear and multiband neural dynamics in MDD and precisely regulate the diseased neural dynamics to therapeutic targets, significantly outperforming open-loop and responsive neuromodulation methods. Our results have implications for building future clinical closed-loop BCI systems for treating MDD.

## Data availability statement

The raw data supporting the conclusions of this article will be made available by the authors, without undue reservation.

## Author contributions

YY conceptualized the study. YY and HF developed the BCI system, the system identification methods, the predictive neuromodulation methods, and wrote the manuscript. HF conducted the simulations and data analyses. Both authors contributed to the article and approved the submitted version.
