# Supplementary material for: Predictive neuromodulation of cingulo-frontal neural dynamics in major depressive disorder using a brain-computer interface system: A simulation study
Source: Front Comput Neurosci. 2023 Mar 6;17:1119685. doi: 10.3389/fncom.2023.1119685 (PMC10025398; doi:10.3389/fncom.2023.1119685)
Supplement: Supplementary file 1 [file Data_Sheet_1.PDF]

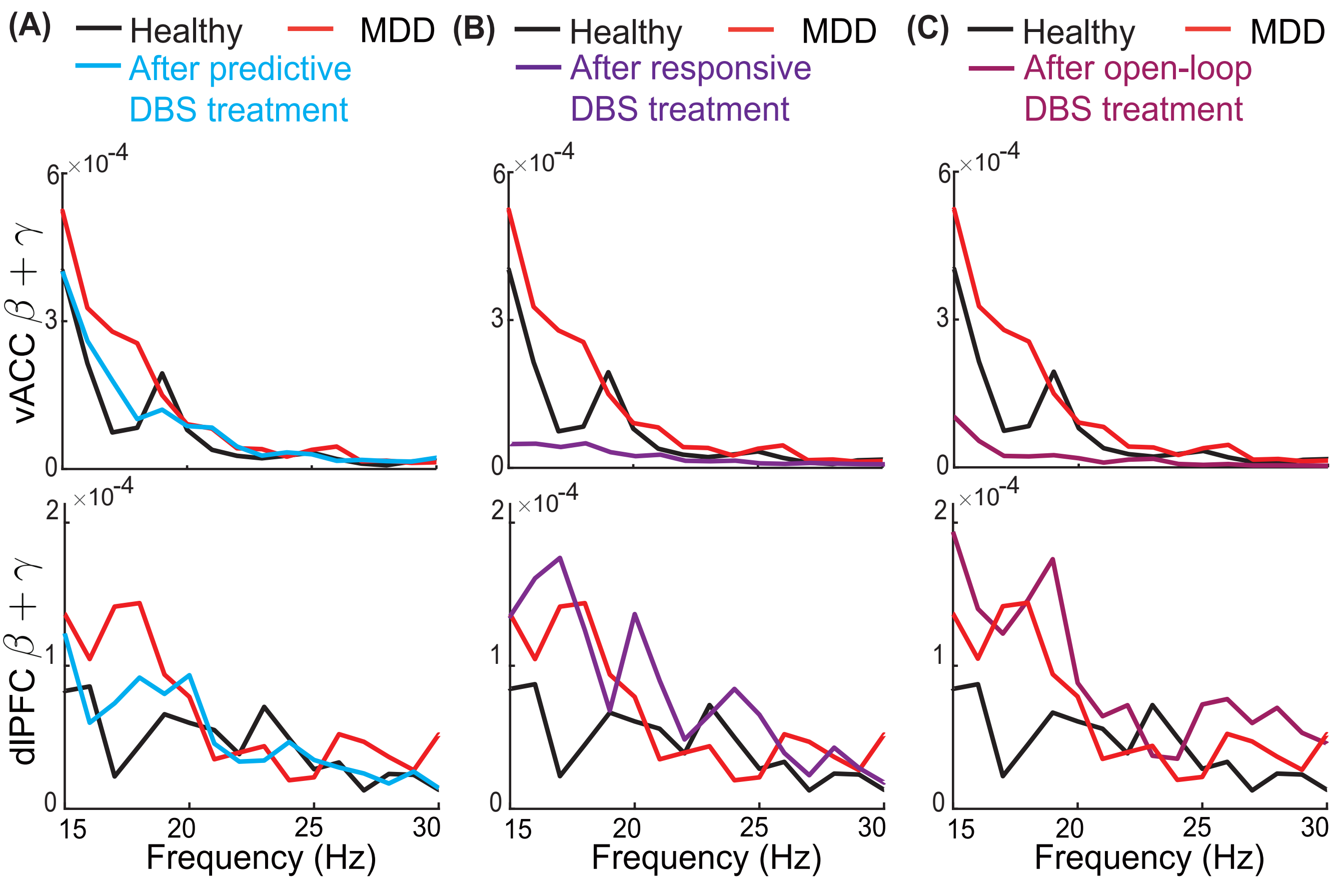

Supplementary figure 1. Changes of $\beta + \gamma$ power spectrum in vACC and dlPFC before and after DBS treatments using linear scales. (A) Changes in vACC (top) and dlPFC (bottom) power spectrum before (red) and after (cyan) predictive DBS treatments. The power spectrum in the healthy state is shown in black. Note that the vertical axis is in linear scale. (B) Same as (A) but for responsive DBS. (C) Same as (A) but for open-loop DBS.

SUPPLEMENTARY FIGURES
